# Identifying candidate genes for *Phytophthora capsici* resistance in pepper (*Capsicum annuum*) via genotyping-by-sequencing-based QTL mapping and genome-wide association study

**DOI:** 10.1038/s41598-019-46342-1

**Published:** 2019-07-10

**Authors:** Muhammad Irfan Siddique, Hea-Young Lee, Na-Young Ro, Koeun Han, Jelli Venkatesh, Abate Mekonnen Solomon, Abhinandan Surgonda Patil, Amornrat Changkwian, Jin-Kyung Kwon, Byoung-Cheorl Kang

**Affiliations:** 1 Department of Plant Science and Plant Genomics and Breeding Institute, Seoul National University, Seoul, 08826 Republic of Korea; 2 National Academy of Agricultural Science, National Agrobiodiversity Center, Rural Development Administration, Jeonju, 54874 Republic of Korea

**Keywords:** Plant hybridization, Plant breeding

## Abstract

*Phytophthora capsici* (Leon.) is a globally prevalent, devastating oomycete pathogen that causes root rot in pepper (*Capsicum annuum*). Several studies have identified quantitative trait loci (QTL) underlying resistance to *P. capsici* root rot (PcRR). However, breeding for pepper cultivars resistant to PcRR remains challenging due to the complexity of PcRR resistance. Here, we combined traditional QTL mapping with GWAS to broaden our understanding of PcRR resistance in pepper. Three major-effect loci (*5.1*, *5.2*, and *5.3*) conferring broad-spectrum resistance to three isolates of *P. capsici* were mapped to pepper chromosome P5. In addition, QTLs with epistatic interactions and minor effects specific to isolate and environment were detected on other chromosomes. GWAS detected 117 significant SNPs across the genome associated with PcRR resistance, including SNPs on chromosomes P5, P7, and P11 that colocalized with the QTLs identified here and in previous studies. Clusters of candidate nucleotide-binding site-leucine-rich repeat (NBS-LRR) and receptor-like kinase (RLK) genes were predicted within the QTL and GWAS regions; such genes often function in disease resistance. These candidate genes lay the foundation for the molecular dissection of PcRR resistance. SNP markers associated with QTLs for PcRR resistance will be useful for marker-assisted breeding and genomic selection in pepper breeding.

## Introduction

Pepper (*Capsicum annuum*) is an economically important horticultural crop grown in tropical and temperate regions that is used as a fresh vegetable and as a processed food product. *Phytophthora capsici* (Leon.) is an invasive oomycete that poses a serious threat to pepper production across the globe^1^. *P. capsici* can infect pepper plants at any growth and developmental stage, causing damping off, root rot, stem rot, collar rot, fruit rot, and foliar blight. *P. capsici* root rot (PcRR) is the most devastating disease, causing up to 100% yield losses under warm (25–28 °C), humid environmental conditions^2^. The pathogen may enter the plant system through the roots or stem collar, causing water-soaked lesion formation and stem girdling, eventually leading to plant wilting and death. The broad range of host species of *P. capsici*, including Solanaceae, Fabaceae, and Cucurbitaceae, as well as its soil-born, random mating nature, makes it quite difficult to control PcRR. Cultural practices and chemical control measures for PcRR have proven to be ineffective and unsafe^3^. The use of cultivars resistant to PcRR represents the best control measure due to its eco-friendliness and cost-effectiveness.

Resistance to PcRR is influenced by several factors, including environmental cues, the virulence of the *P. capsici* isolates, their physiological races, and the source of resistance^2,4^. *P. capsici* isolates, with their short life cycles and rapid evolution of virulence, have a selective advantage over their slowly evolving plant hosts in the evolutionary arms race^5,6^. More than 45 physiological races have been described within the *Phytophthora* for each host plant species^7^. Three physiological races of *P. capsici* were identified in four pepper cultivars in Taiwan^7^, and recently, 24 new races were identified using recombinant inbred lines^8^. The virulence of *P. capsici* isolates is another factor that may affect the genetic analysis of resistance mechanisms. The presence of isolates with low virulence could lead to false resistance, whereas highly virulent isolates could overcome the resistance of plant lines^9^. Several resistance sources have been reported, including *C*. *annuum* lines CM334, PI201232, PI201234, AC2258, Perennial, and YCM334^4,9,10^. These resistant sources have been reported with varying levels of resistance to each *P. capsici* isolate^11^. Among these sources, CM334 has the highest resistance and has been extensively utilized in breeding programs^12^. Nevertheless, breeding for PcRR resistance is a complex process, and the resistance levels of commercial pepper cultivars are not comparable to those of the original resistance sources^2,11^. Therefore, the genetic improvement of pepper for increased resistance to PcRR is still an important objective of most pepper breeding programs.

In Solanaceae, resistance to *Phytophthora* is race specific and quantitative in nature^13–15^. The colocalization of single resistance genes and QTLs for resistance to *Phytophthora* has been reported in various Solanaceous species, indicating that homologous resistance genes control resistance to *Phytophthora*^16^. Two race-specific resistance genes have been reported in pepper: *CaPhyto* from PI201234 and *PhR10* from CM334 on chromosomes P5 and P10, respectively^9,17^. In addition, major and minor resistance QTLs to PcRR have been mapped onto various chromosomes^11,18,19^. Major resistance QTLs to PcRR have been consistently identified in close proximity on chromosome P5 in various studies irrespective of the resistance sources or *P. capsici* isolates^4,10,18,20,21^. Based on meta-analysis of QTLs, the major effect QTL, *MetaPc5.1* was localized to the 22.4–24.6 Mb region on chromosome P5, and *MetaPc5.2* and *MetaPc5.3* were localized to the 53.0–162.6 Mb and 9.7–13.3 Mb regions on chromosome P5, respectively^9,21^. Several minor and isolate-specific QTLs have been reported, but their positions are variable, depending on the genetic background and *P. capsici* isolate^4,19,22^. Several molecular markers linked to the major QTLs, such as Phyto5NBS1 and ZL6726, have been developed and utilized for marker-assisted selection (MAS) in pepper, but their use is limited to the selection of resistant plants to highly virulent and race nonspecific *P. capsici* isolates^9,10^.

Ultra-high-density linkage maps play a crucial role in genetic and genomics analyses. The emergence of next-generation sequencing (NGS) technologies and high-throughput genotyping tools has facilitated the rapid discovery of SNP markers. Among the numerous NGS technologies, genotyping by sequencing (GBS) is a simple, efficient, rapid approach with the potential to detect polymorphisms at relatively low cost. GBS has been widely used in several crops for biparental QTL mapping and genome-wide association study (GWAS) to uncover loci controlling various traits^23–25^.

Previous genetic studies of PcRR resistance in pepper have mainly focused on biparental QTL mapping, which primarily depends on the genetic diversity of the two parental lines. Therefore, the detected QTLs encompass large genomic regions with low map resolution, thus hampering the development of tightly linked markers and the identification of candidate genes^10,24^. These limitations could be overcome by employing GWAS, which allows QTL regions to be narrowed down to the candidate gene level using natural populations. However, GWAS has a high rate of false-positive QTL detection, requiring additional validation^26,27^. The shortcomings of biparental QTL mapping and GWAS can be overcome by combining these two approaches^24^. Such an approach had been successfully utilized for the genetic dissection of several important plant traits, including frost resistance, flowering time, panicle architecture, leaf architecture, pungency, and seed-related traits^24,28–32^.

In this study, we developed F_7:8_ recombinant inbred lines (RILs) derived from a cross between resistance source *C*. *annuum* cv. CM334 and susceptible cv. ECWR30 for QTL analysis of PcRR resistance to three *P. capsici* isolates in two environments. We then used previously reported core collections^33^ to evaluate PcRR resistance via GWAS. We used high-density SNP markers developed by GBS to identify and validate novel QTLs associated with PcRR resistance. By combining GWAS with QTL analysis, we successfully identified candidate genes for PcRR resistance.

## Results

### Evaluation of PcRR resistance in ECRILs and the GWAS core collection

**Figure 1:**
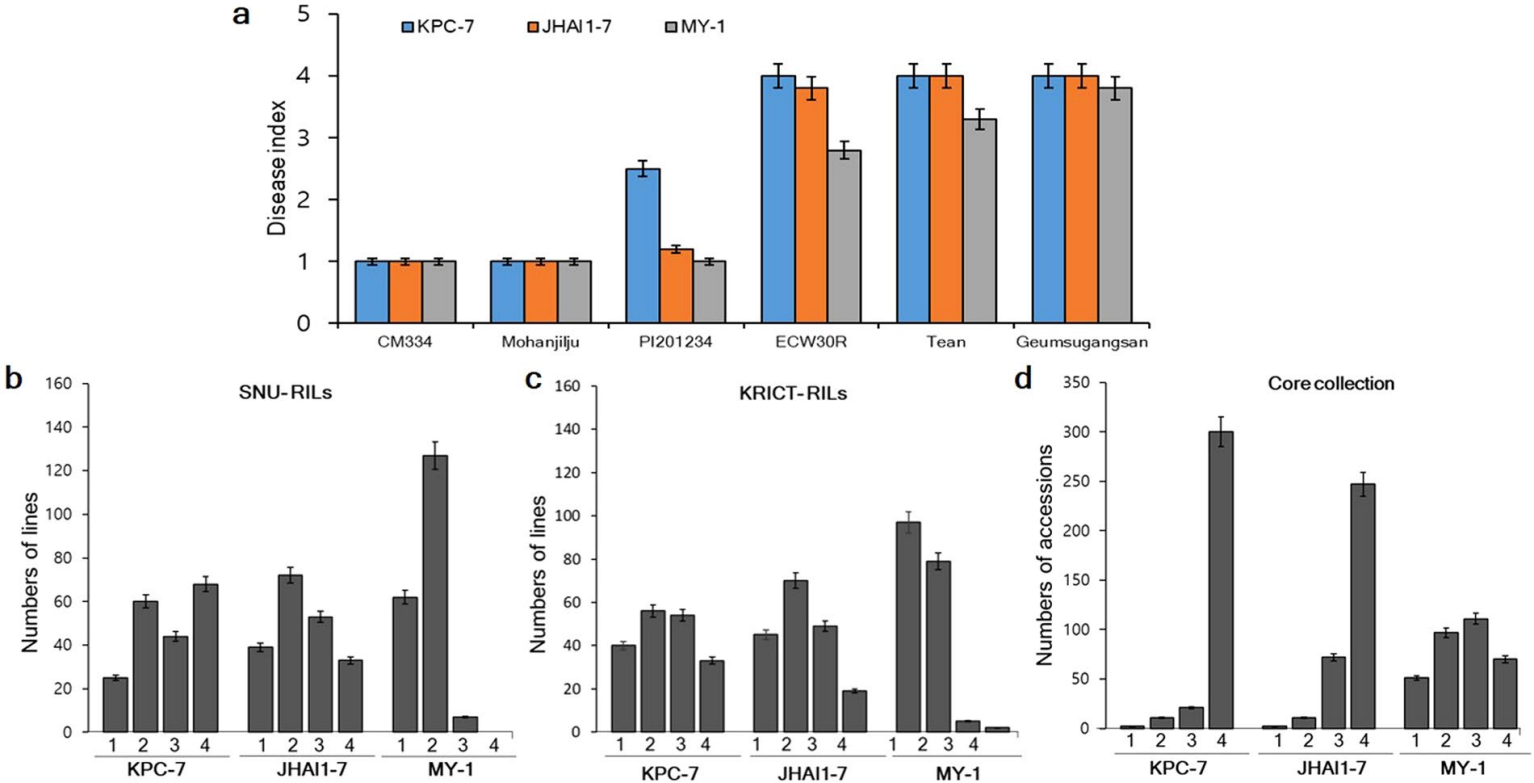
Frequency distribution of PcRR in the parental lines, controls, ECRILs (SNU-RILs/KRICT-RILs), and GWAS (core collection). (**a**) Disease index of resistant controls (cv. CM334, Mohanjilju, and PI201234) and susceptible parents (cv. ECW30R, Tean, and Geumsugangsan) at three weeks post inoculation. (**b**) Frequency distribution of ECRILs against isolates KPC-7, JHAI1-7, and MY-1 in environment E1 (SNU-RILs). (**c**) Frequency distribution of ECRIL against isolates KPC-7, JHAI1-7, and MY-1 in environment E2 (KRICT-RILs). (**d**) Frequency distribution of GWAS (core collection) against isolates KPC-7, JHAI1-7, and MY-1. Bars denote the standard deviation.

We evaluated 188 F_7:8_ ECRILs, parental lines, and additional resistant and susceptible controls in two environments (E1 and E2) for resistance to three *P. capsici* isolates (Fig. 1). The three isolates showed a significant difference in terms of symptom development and virulence. Susceptible controls (ECW30R, Tean, and Geumsugangsan) infected with the highly virulent isolate KPC-7, showed disease symptoms, including wilting and the appearance of water-soaked lesions at stem collars within 72 h of inoculation, and the plants completely wilted and died at 7 to 10 days post inoculation (DPI). By contrast, in plants infected with the moderately virulent isolate JHAI1–7, we noticed disease symptoms on the susceptible controls at 5 to 7 DPI, and the plants completely wilted and died at two weeks post inoculation. Susceptible controls infected with the isolate MY-1, with low virulence, symptoms were observed at 10 to 14 DPI, but complete plant death was not detected up to three weeks post inoculation. No visual symptoms of collar rot were observed on resistant controls cv. CM334 and Mohanjilju (commercial F_1_ hybrid, Syngenta Korea) after inoculation with all three isolates, even at three weeks post inoculation (Fig. 1a). However, PI201234 was partially resistant to isolates KPC-7 and JHAI1-7 and completely resistant to isolate MY-1 (Fig. 1a).

We evaluated PcRR resistance in the ECRIL and GWAS core collection and scored disease severity on a scale of 1–4 at three weeks post inoculation in their respective environments, as described by^36^ (Supplementary Fig. S1). Ten plants from each ECRIL were grown and the resistance level of each plant scored individually. We calculated the frequency distribution based on the mean resistance values of each RIL (Fig. 1b,c). The frequency distribution of disease severity showed a normal distribution for KPC-7 and JHAI-7 in both environments, although more ECRILs showed susceptibility to KPC-7 in E1 than E2 (Fig. 1b,c). By contrast, the distribution of disease severity of the RILs skewed towards resistance to the low-virulence isolate MY-1, and no ECRILs reached the disease rating of 4 during the experiment in either environment, except for two ECRILs in environment E2 (Fig. 1b,c and Supplementary Fig. S2). Correlation analysis indicated a significant, positive correlation between the two environments for PcRR resistance; however, the correlations between different isolates varied (Supplementary Fig. S2).

We evaluated the GWAS accessions for PcRR resistance against the same three isolates as the ECRILs in two different environments (EA and E1). Among GWAS accessions, CM334 was completely resistant, whereas PI201234, YCM334, and nine other accessions were partially resistant, with slight disease symptoms at the stem collars upon infection with isolates KPC-7 and JHAI-7 (Fig. 1a,d and Supplementary Table S2). Most of these PcRR-resistant accessions include known resistance sources (Supplementary Table S2). The isolate JHAI1-7 showed slightly higher virulence compared to KPC-7 in a few lines of GWAS core collection, but a higher mortality rate was observed for KPC-7 (Fig. 1d and Supplementary Fig. S3). The disease response to the low-virulence isolate MY-1 was less severe, with 52 accessions showing no PcRR symptoms (Fig. 1d). Correlation analysis revealed significant, positive correlations between the KPC-7 and JHAI1-7 isolates and both environments for PcRR resistance (Supplementary Fig. S3). Highly virulent isolate KPC-7 for ECRILs in E2 and moderately virulent isolate JHAI1-7 for the GWAS accessions showed a minimum coefficient of variation (CV) of 38.55 and 28.09%, respectively (Table 1). Weak isolate MY-1 showed the highest (CV 57.0 to 62.02%) disease score in both screened populations in all environments (Table 1). The broad-sense heritability (H^2^%) was found to be high for all PcRR isolates. In E1, isolate KPC-7 showed the highest H^2^ (82.48%) among ECRILs, whereas isolate JAHI-7 showed an H^2^ of 87.40% and 84.16% for ECRILs in E2 and the GWAS accessions, respectively (Table 1).

**Table 1:** Genetic variation among ECRILs and GWAS accessions inoculated with three isolates of *P. capsici* in different environments/years.

Population (Environment-year)	Isolate	Mean ± SE	Range (Max-Min)	CV (%)	H² (Broad-sense) (%)	Expected genetic advancement (5%)
SNU-ECRILs (E1-2017)	KPC-7	2.12 ± 0.28	4–1	42.05	82.48	170.75
	JHAI-7	1.75 ± 0.25	4–1	46.10	80.22	171.30
	MY-1	1.16 ± 0.21	2.09–1	57.00	52.45	89.32
KRICT-ECRILs (E2-2018)	KPC-7	2.38 ± 0.91	4–1	38.55	80.21	153.17
	JHAI-7	2,19 ± 0.84	4–1	38.60	87.40	124.16
	MY-1	1.43 ± 0.36	4–1	56.29	75.82	178.83
GWAS Population (EA-2017, E1-2018)	KPC-7	2.86 ± 0.45	4–1	50.09	59.61	96.79
	JHAI-7	3.03 ± 0.26	4–1	28.09	84.16	122.39
	MY-1	1.89 ± 0.37	4–1	62.06	72.85	178.73

### SNP discovery through GBS and bin map construction for ECRILs

**Figure 2:**
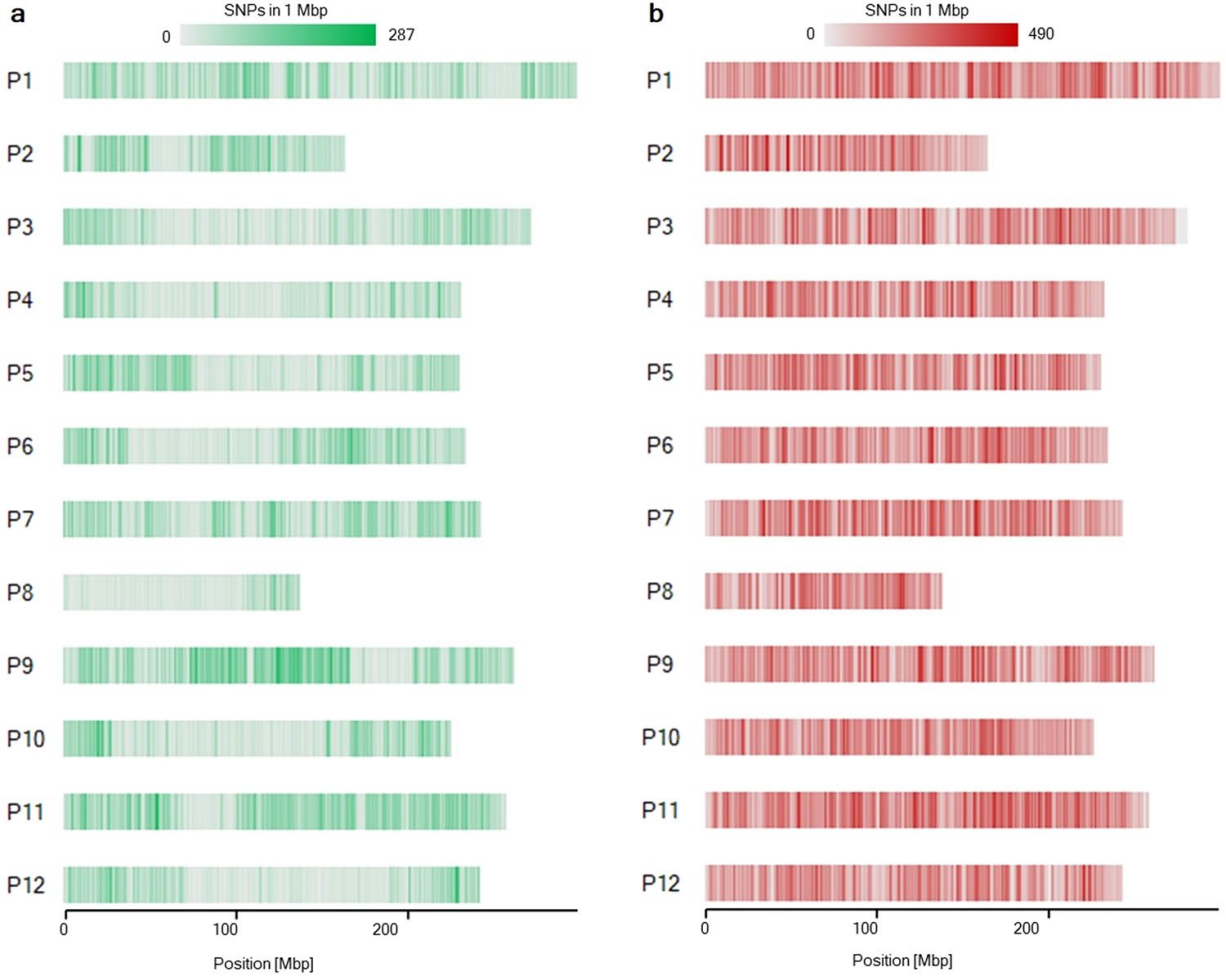
SNP density (number of SNPs per 1 Mbp). (**a**) SNP density for ECRILs digested with *Eco*RI/*Mse*I. (**b**) SNP density for the GWAS (core collection) digested with *Pst*I/*Mse*I and *Eco*RI/*Mse*I.

We performed genotyping of the ECRILs using GBS after *Eco*RI and *Mse*I digestion. Sequencing of the prepared GBS libraries of 188 ECRILs and parental lines resulted in 525.62 million raw reads (data not shown). After trimming the raw reads, we obtained an average of approximately 4.4 million reads per sample (Table 2). After aligning the reads to the CM334 reference genome v1.6, 66,405 SNPs were detected (Fig. 2a). The SNPs were distributed more densely at the ends of the chromosomes than in the middle regions (Fig. 2a). After removing more than 90% of missing data and filtering unequally distributed SNPs, we obtained 13,021 high-quality SNPs that were equally distributed across the genome, which were used for bin linkage map construction (Tables 2 and 3 and Supplementary Fig. S4). To impute missing data and genotyping errors, we employed a sliding window approach^23^. Recombination breakpoints were determined by sliding 25 SNPs consecutively as one window. As a result, a high-density bin map consisting of 2,663 bins covering a total genetic distance of 1,428.8 cM was constructed (Table 3 and Supplementary Fig. S4). The average genetic distance between bins was estimated to be 0.6 cM (Table 3). Among the 12 linkage groups, maximum and minimum genetic distances of 195.6 and 44.7 cM were obtained for chromosomes P1 and P8, respectively (Table 3).

**Table 2:** Genotyping data for GWAS and QTL mapping.

	ECRIL	GWAS core collection
Number of accessions (lines)	188	352
Genotyping method	GBS (*Eco*RI/*Mse*I)	GBS (*Pst*I/*Mse*I and *Eco*RI/*Mse*I)
Average number of reads per sample	4,450,271*	3,326,422
Total number of retained SNPs	13,021	507,713
Average distance between SNPs (bp)	366,349	16,305

*Number of spots sequenced by paired-end reads.

**Table 3:** Comparison of the physical length and genetic distance of bins for biparental QTL mapping.

Chr.	Number of SNPs	Number of bins	Physical length of bin (Mb)		Genetic distance of bin (cM)	
			Mean	Total	Mean	Total
P1	1551	329	1.2	272.7	0.6	195.6
P2	925	190	1.0	171.1	0.5	94.1
P3	887	275	1.1	257.9	0.6	162.9
P4	613	191	1.5	222.6	0.6	125.6
P5	1009	183	1.2	233.5	0.5	91.6
P6	1034	219	1.1	236.9	0.5	114.4
P7	1323	259	1.1	231.9	0.5	129.4
P8	212	52	2.1	145.1	0.9	44.7
P9	1864	260	1.2	252.8	0.5	119
P10	808	207	1.3	233.6	0.5	115.3
P11	2127	323	0.9	259.7	0.4	116.1
P12	668	175	1.2	235.7	0.7	120.1
Total	13,021	2,663	1.2	2,753.5	0.6	1,428.8

### PcRR resistance QTLs in the ECRILs

**Figure 3:**
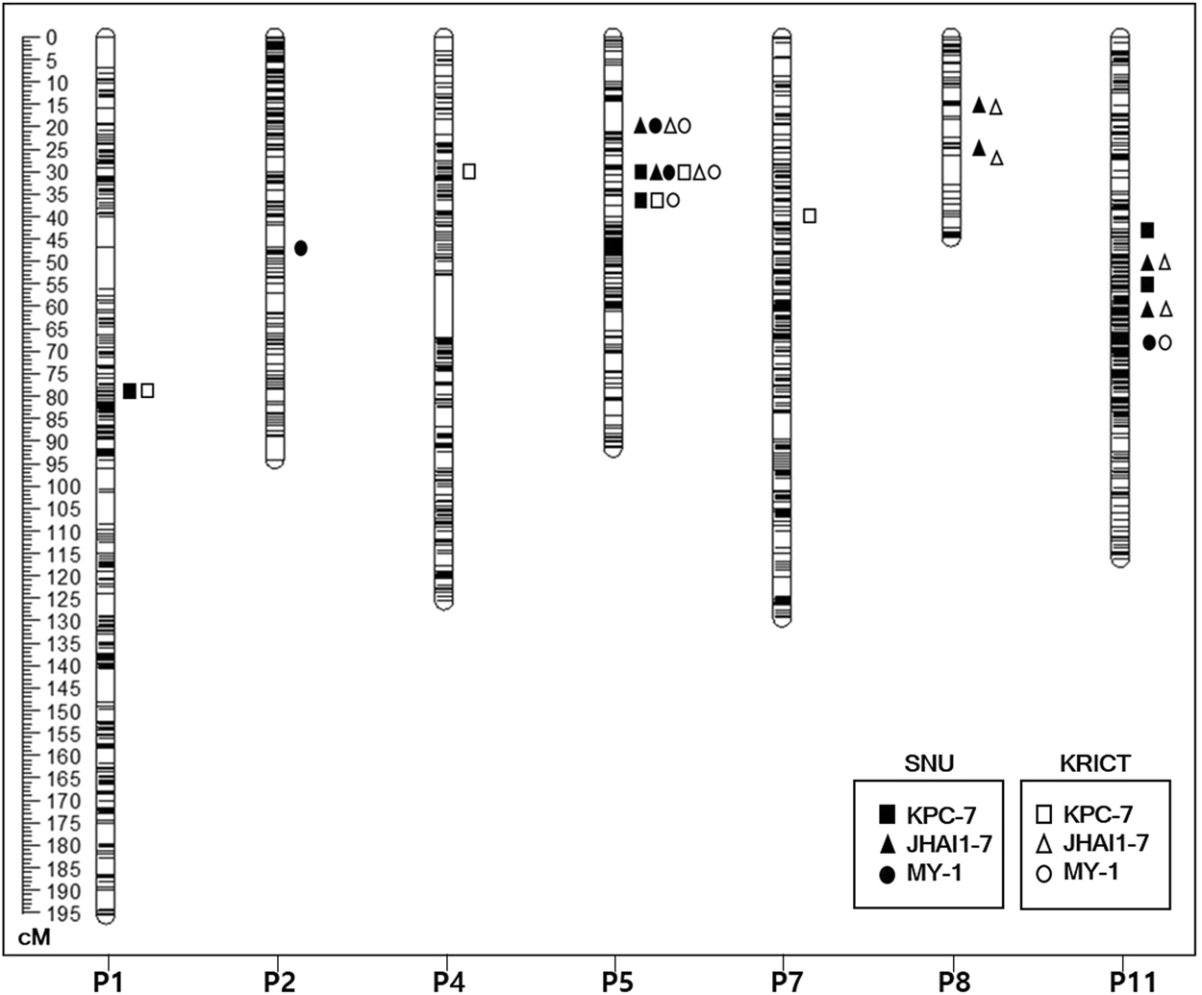
Bin linkage chromosomal map showing the locations of PcRR resistance QTLs with the genetic distance shown in centimorgans (cM) for the ECRIL population evaluated with three *P. capsici* isolates in two respective environments.

We identified QTLs for PcRR resistance to three isolates, KPC-7, JHAI1-7, and MY-1, using 188 ECRILs and the high-density SNP linkage map in two environments, E1 and E2 (Fig. 3 and Table 4). In total, 14 QTLs, including those with major and minor effects, those commonly detected, those specific to isolates, and those specific to environments were detected across the genome, explaining phenotypic variation (R^2^) ranging from 3.73 to 38.99% (Table 4). The PcRR resistance QTLs were detected on chromosomes P1, P2, P4, P5, P7, P8, and P11.

**Table 4:** PcRR resistance QTLs detected by composite interval mapping in the ECRIL population inoculated with three isolates evaluated in their respective environments.

Isolate	Evaluating environment	QTLs	Chr.	Position^a^ (cM)	Location^b^ (Mb)	LOD^c^	R^2^ (%)^d^	Additive effect
KPC-7	E1-SNU*2017	*E1Kpc-1*	P1	77.61	9.5–9.63	3.97	4.57	2.27
		*E1Kpc-5.2*	P5	28.91	27.3–29.2	21.32	33.77	6.26
		*E1Kpc-5.3*	P5	34.61	34.6–37	14.87	25.40	5.41
		*E1Kpc-11.1*	P11	41.40	49.5–50.5	4.78	5.59	2.53
		*E1Kpc-11.2*	P11	52.01	74–76	4.20	4.94	2.40
	E2-KRICT*2018	*E2Kpc-1*	P1	77.61	9.5–9.63	6.69	7.81	4.42
		*E2Kpc-4*	P4	28.41	87–88.5	4.64	5.41	2.96
		*E2Kpc-5.2*	P5	29.21	27.3–29.2	23.25	37.12	5.78
		*E2Kpc-5.3*	P5	34.61	34.6–37	14.17	25.14	4.70
		*E2Kpc-7*	P7	38.71	24.5–26.4	3.60	3.73	1.80
JHAI1–7	E1-SNU*2017	*E1Jha-5.1*	P5	22.61	18.7–19.5	18.30	27.31	4.80
		*E1Jha-5.2*	P5	28.91	27.3–29.2	29.73	38.99	5.72
		*E1Jha-8.1*	P8	13.31	126.4–126.6	7.25	6.85	2.40
		*E1Jha-8.2*	P8	23.71	128.2–130.3	5.82	5.27	2.06
		*E1Jha-11.1*	P11	48.41	61.2–63	9.01	8.47	2.62
		*E1Jha-11.2*	P11	59.31	111.4–112.8	7.71	7.37	2.47
	E2-KRICT*2018	*E2Jha-5.1*	P5	22.61	18.7–19.5	18.13	26.62	4.54
		*E2Jha-5.2*	P5	28.91	27.3–29.2	29.66	38.21	5.44
		*E2Jha-8.1*	P8	12.31	126.4–126.6	4.61	4.36	1.83
		*E2Jha-8.2*	P8	19.51	128.2–130.3	4.57	4.26	1.77
		*E2Jha-11.1*	P11	48.41	61.2–63	9.44	8.82	2.57
		*E2Jha-11.2*	P11	60.51	111.4–112.8	7.34	7.03	2.33
MY-1	E1-SNU*2017	*E1My-2*	P2	45.91	129–131	4.30	6.99	1.00
		*E1My-5.1*	P5	22.61	18.7–19.5	8.94	18.22	1.60
		*E1My-5.2*	P5	28.61	27.3–29.2	10.80	19.68	1.66
		*E1My-11*	P11	66.41	189–190	4.89	8.13	1.07
	E2-KRICT*2018	*E2My-5.1*	P5	22.31	18.7–19.5	4.97	10.02	1.19
		*E2My-5.2*	P5	28.61	27.3–29.2	6.36	12.61	1.35
		*E2My-5.3*	P5	34.61	34.6–37	4.38	8.90	1.12
		*E2My-11*	P11	64.41	189–190	3.33	6.17	1.50

^a^Positions of the markers on the linkage map in centimorgans (cM).

^b^Position of right and left bins in the pepper genome. ^c^Maximum log-likelihood (LOD) value of QTL. ^d^Phenotypic variation (R^2^) explained by a QTL at the linked marker.

*E1-SNU, Environment 1, Seoul National University; *E2-KRICT, Environment 2, Korea Research Institute of Chemical Technology.

Two major QTLs to highly virulent isolate KPC-7 were detected on chromosome P5, *E1Kpc-5.2* and *E2Kpc-5.3*, at 28.91 and 34.61 cM, corresponding to 27.3–29.2 Mb and 34.6–37 Mb, respectively, in the *C*. *annuum* ‘CM334’ reference genome. *E1Kpc-5.2* explained 33.77% (LOD of 21.32) and 37.12% (LOD of 23.25) and *E2Kpc-5.3* explained 25.40% (LOD of 14.87) and 25.14% (LOD of 14.17) of phenotypic variation (R^2^) in E1 and E2, respectively (Fig. 3 and Table 4). Three minor QTLs were detected in both environments, but only one QTL (on chromosome P1 at 77.61 cM [9.5–9.6 Mb]) was commonly detected in both environments (Fig. 3 and Table 4).

Two major QTLs to moderately virulent isolate JHAI1–7 on chromosome P5, *E1Jha-5.1* and *E2Jha-5.2*, were detected at 22.61 and 28.91 cM, corresponding to 18.7–19.5 and 27.3–29.2 Mb, respectively, in the *C*. *annuum* ‘CM334’ reference genome. *E1Jha-5.1* explained 27.31% (LOD of 18.30) and 26.62% (LOD of 18.13) and *E2Jha-5.2* explained 38.99% (LOD of 29.73) and 38.21% (LOD of 29.66) of phenotypic variation (R^2^) in E1 and E2, respectively (Fig. 3 and Table 4). Four minor QTLs on P8 and P11 were detected in both environments (Fig. 3 and Table 4).

For low-virulence isolate MY-1, two major-effect QTLs on chromosome P5, *E1My-5.1* and *E2My-5.2*, were detected at 22.61 and 28.61 cM, corresponding to 18.7–19.5 Mb and 27.3–29.2 Mb, respectively, in the *C*. *annuum* ‘CM334’ reference genome. *E1My-5.1* explained 18.22% (LOD of 8.94) and 10.02% (LOD of 4.94) and *E2My-5.2* explained 19.68% (LOD of 10.80) and 12.61% (LOD of 6.33) of phenotypic variation (R^2^) in E1 and E2, respectively (Fig. 3 and Table 4). One major QTL on chromosome P5, *E1My-5.3*, was detected in E2 only at 34.61 cM, corresponding to 34.6–37 Mb, which explained 8.90% (LOD 4.38) of phenotypic variation (R^2^). Two minor QTLs were detected in both environments, but only one minor QTL was commonly detected in both environments (on chromosome P11 at 66.41 cM [189–190 Mb]; Fig. 3 and Table 4). Notably, all QTLs associated with the MY-1 isolate showed lower R^2^ and LOD values when compared to the QTLs detected against the two other isolates used in this study (Fig. 3 and Table 4).

Taken together, three major QTLs on P5 were commonly detected for all three isolates in two environments, whereas minor QTLs on different chromosomes were isolate or environment specific (Fig. 3 and Table 4). Among the major QTLs, *QTL5.2*, which is located at 27.3–29.2 Mb, was commonly detected for all three isolates and was named according to its genomic position on chromosome P5. The major QTL at 18.7–19.5 Mb (common to JHAI1–7 and MY-1) was named *QTL5.1*. The major QTL at 34.6–37 Mb (common to KPC-7 and MY-1) was named *QTL5.3*. Even though minor QTLs were commonly detected on chromosome 11 for all three isolates, they were located at different positions. We detected isolate-specific minor QTLs on other chromosomes, such as the minor QTLs *Kpc1* (for KPC-7) on P1, *Jha8.1*, *Jha8.2*, *Jha11.1*, and *Jha11.2* (for JHAI1-7) on P8 and P11, and *My11* (for MY-1) on P11.

### Epistatic interactions of PcRR resistance QTLs

We detected additive-by-additive epistatic interactions against highly virulent isolate KPC-7 between major QTLs *QTL5.2* and *QTL5.3* and *QTL5.2* and *E1Kpc-11.1* in environment E1 and between QTLs *QTL5.2* and *QTL5.3* in environment E2 (Supplementary Table S3). We detected additive-by-additive epistatic interactions between *QTL-5.2* and *Jha8.1* and *QTL5.2* and *Jha11.2* in environment E1 and between QTLs *QTL5.2* and *Jha11.1* in environment E2 (Supplementary Table S3) against moderately virulent isolate JHAI1-7. By contrast, we detected additive-by-additive epistatic interactions only between *QTL5.2* and *My11* in both E1 and E2 (Supplementary Table S3) against low-virulence isolate. The total R^2^ effects of individual QTLs and their interactions ranged from 16.6 to 42.0% (Supplementary Table S3). The detection of epistatic interactions between loci on chromosomes P5 and P11 points to multilocus epistatic control of PcRR resistance in pepper.

### Genome-wide association study of PcRR resistance

We aligned the sequences derived from GBS to *C*. *annuum* cv. CM334 reference genome v.1.6. GBS genotyping of 352 accessions using two sets of libraries derived from double digestion with restriction enzyme pairs *Pst*I/*Mse*I and *Eco*RI/*Mse*I generated a total of 6,126,403 of SNPs. The SNPs were evenly distributed using this combination of restriction enzymes. SNPs in the GWAS core collection were relatively uniformly distributed across the chromosomes (Fig. 2b). We filtered out the SNPs with minor allele frequency >0.05, SNP coverage >0.6, and inbreeding coefficient >0.8, resulting in 507,713 high-quality SNPs distributed evenly on chromosomes P1 to P12 (Fig. 2b and Table 2).

**Figure 4:**
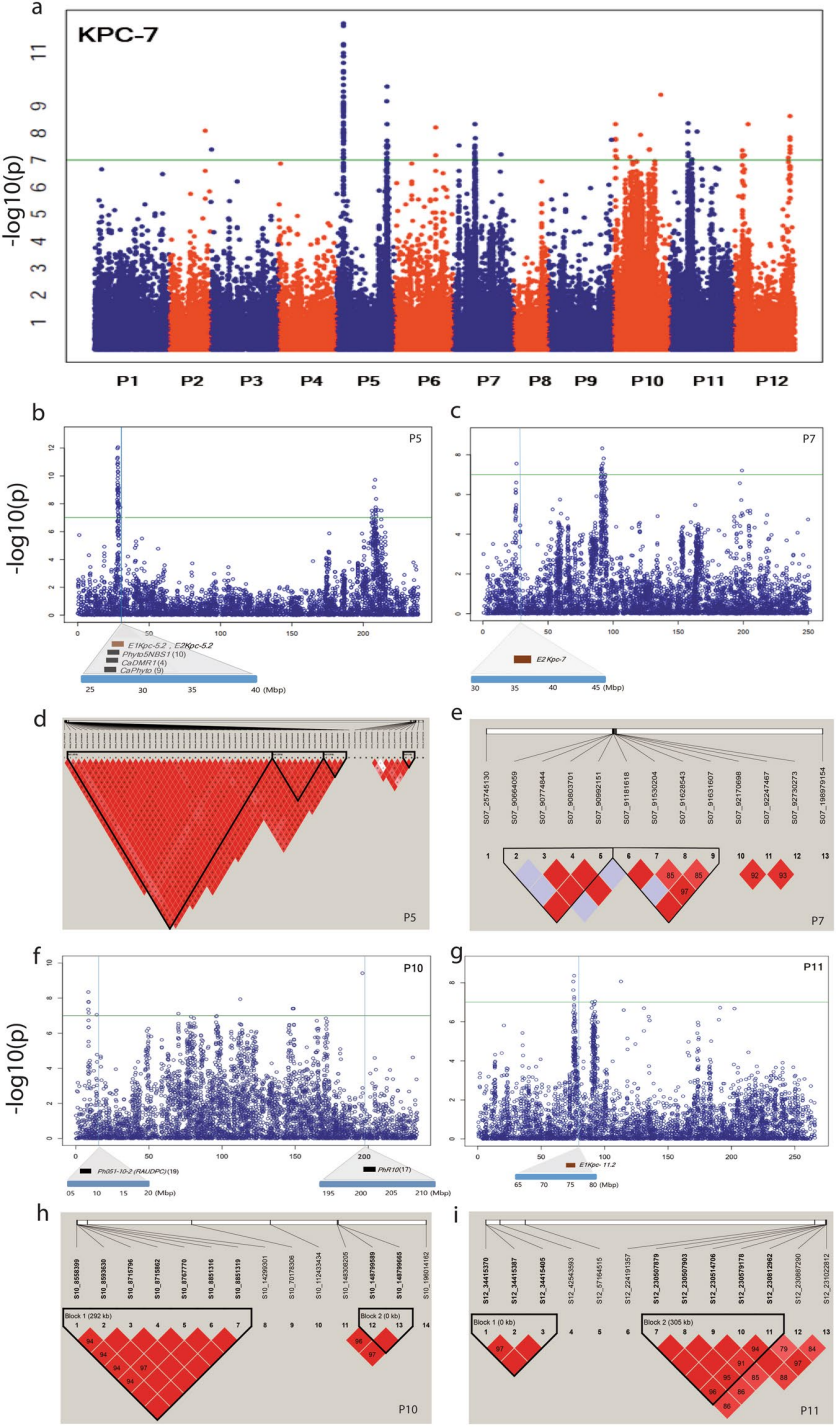
Manhattan plots based on GBS-GWAS showing the significant SNPs associated with PcRR resistance and haplotype analysis. (**a**) Significant SNPs associated with PcRR isolate KPC-7. (**b**,**c**,**f**,**g**) SNPs detected in common regions of GWAS and ECRIL QTL maps from this and previous studies. QTL positions are marked with blocks under the SNP positions. Brown and black blocks indicate QTLs detected in this and previous studies, respectively. (**d**,**e**,**h**,**i**) Haplotype blocks containing significant SNPs on chromosomes P5, P7, P10, and P11.

Of the SNPs association with resistance to PcRR against isolate KPC-7, 117 were significant SNPs, with -log10 (p) value > 7.0, explaining phenotypic variation (R^2^) > 0.2 (Fig. 4a and Supplementary Table S4). An association study revealed significant SNPs linked to PcRR resistance to KPC-7 on chromosomes P2 (1 SNP at 149 Mb), P3 (1 SNP at 5 Mb), P5 (64 SNPs at 27.7–29.7 and 206–212 Mb), P6 (2 SNP at 171 Mb), P7 (13 SNPs at 24 and 90 Mb), P9 (1 SNP at 263 Mb), P10 (14 SNPs at 8, 14, 70, 112, 148, and 196 Mb), P11 (8 SNPs at 75, 90, 92, and 112 Mb), and P12 (13 SNPs at 34, 42, 57, 224, and 230 Mb) (Fig. 4a and Supplementary Table S4). GWAS of isolate JHAI1-7 revealed an associated SNP peak on chromosome P5 with a similar position to that of the KPC-7 isolate (27.7 to 29.7 Mb), but only two SNPs showed a significance level above the adjusted threshold level at -log10(p) value > 7.0, explaining phenotypic variation (R^2^) > 0.2 (Supplementary Fig. S5a and Supplementary Table S4). The two significant SNPs were located at 28.2 to 28.6 Mb on chromosome P5. By contrast, GWAS against the low-virulence isolate MY-1 did not detect significant SNPs (Supplementary Fig. S5b). Notably, only a few or no significant SNPs were associated with isolates JHAI1-7 and MY-1, perhaps due to environment and genotype interactions or to the low virulence levels of the isolates.

Importantly, significant SNPs associated with resistance to isolate KPC-7 on three regions of chromosomes P5, P7, and P11 colocalized with QTLs detected in this study (Fig. 4a and Table 4). On chromosome P5, 49 SNPs colocalized with commonly detected major QTL region *QTL5.2* (27.3–29.2 Mb) (Fig. 4b and Table 4). One SNP detected on chromosome P7 at 25 Mb corresponded to the location of QTL *E2Kpc-7* (Fig. 4c and Table 4). Another region on chromosome P11 at 75.2 Mb corresponded to QTL *E1Kpc-11.2* (Fig. 4g and Table 4). The GWAS-SNPs at three regions on chromosomes P5, P10, and P11 colocalized with previously detected QTLs and linked markers (Fig. 4b,f). The GWAS-SNPs on chromosome P5 in a 2.5-Mb region at position 27.0–29.5 Mb colocalized with a QTL detected in earlier reports^4,9,10,21^ (Fig. 4b). SNP marker S10_14299301 located at 14,299 kb on chromosome P10 colocalized with the previously identified QTL *Ph051-10-2* (*RAUDPC*) for PcRR resistance^19^ (Fig. 4f). Regions detected by GWAS with significant SNPs on chromosome P10 at 196 Mb (Fig. 4f) colocalized with the region containing *PhR10*, a race-specific PcRR resistance locus^17^. We identified 13 significant GWAS-SNPs linked to PcRR resistance at the end of the long arm (206–212 Mb) of chromosome P5 that were not detected in previous studies, emphasizing the involvement of this genomic region in PcRR resistance (Fig. 4a). We used the significant SNP S05_208290460, with a -log10(p) value of 9.72 identified from the GWAS-SNPs (Supplementary Table S4), to compare the resistance levels of the GWAS accessions. As shown in the box plots in Supplementary Fig. S6e, the homozygous genotype (S05_208290460) AA is associated with an increased resistance level against all three isolates compared to the alternative homozygous genotype GG.

We conducted extensive linkage disequilibrium (LD) analysis of the GWAS core collection (N = 352) based on all adjacent marker pairs within a chromosome or within a haplotype block (Supplementary Table S5). We identified 29,269 haplotypes, with an average of 2,439 per chromosome. The average haplotype was 81.2 kb in size, with an average of 16.3 SNPs per haplotype (Table 2 and Supplementary Table S5). Further haplotype block analysis carried out with significant SNPs on chromosome P5 revealed 4 blocks containing 64 significant SNPs (Fig. 4d and Supplementary Table S4). The three blocks on P5 colocalized with *QTL 5.2* detected in this study and with previously identified QTLs^4,9,10,21^ (Fig. 4b,d). Two haplotype blocks were obtained on each of the chromosomes P7, P10 and P11, including regions with significant SNPs (Fig. 4e,h,i). These results indicate that the identified haplotype regions are strongly associated with the PcRR resistance.

### Predicted candidate genes for PcRR

We looked for candidate PcRR resistance genes in the 1 Mb upstream and downstream flanking regions of all significant GWAS-SNPs and bin markers linked to PcRR QTLs. Detailed descriptions of the predicted genes in the target regions are provided in Supplementary Table S6. A major and colocalized genomic region detected for isolate KPC-7 via GWAS and biparental QTL mapping (*QTL5.2*) associated with PcRR resistance encompassing a 2.5 Mb region (27.0–29.5 Mb) (Fig. 3 and 4a) contains 20 predicted genes, including genes for three receptor-like kinase (RLK) domain-containing proteins, two nucleotide-binding site-leucine-rich repeat (NBS-LRR) domain-containing proteins, and one SAR8.2 precursor protein known to be associated with disease resistance (Table 5). Two NBS-LRR genes (*PHT81215.1* and *PHT81216.1*) are located 618 and 319 kb upstream of the most significant SNP (S05_27703815), respectively. The most closely located RLK gene (*PHT81221.1*) was identified 144 kb upstream of a highly significant GWAS SNP. Two RLK genes (*PHT81227.1* and *PHT81229.1*) were identified at 5.5 and 12.4 kb downstream of GWAS (SNP S05_28163627). One candidate gene, encoding the SAR8.2 precursor protein, was detected 148 kb upstream of GWAS SNP (S05_28825990) (Table 5 and Supplementary Table S6). These genes were detected by both QTL mapping and GWAS analysis, making them strong candidate genes for PcRR resistance.

**Table 5:** Candidate resistance genes associated with the significant GWAS regions and QTLs for PcRR and their Gene Ontology (GO) descriptions.

Chr.	Mapping strategy (GWAS/QTL)	Location (bp)	Peptide ID^a^	GO description
P5	GWAS-*QTL5.2*	27085614–27086321	PHT81215.1	PREDICTED: disease resistance protein RPP13-like
P5	GWAS-*QTL5.2*	27311924–27314491	PHT81216.1	PREDICTED: disease resistance protein RPP13-like
P5	GWAS-*QTL5.2*	27558828–27565702	PHT81221.1	Inactive leucine-rich repeat receptor-like serine/threonine-protein kinase
P5	GWAS-*QTL5.2*	28168701–28169191	PHT81227.1	PREDICTED: probable LRR receptor-like serine/threonine-protein kinase
P5	GWAS-*QTL5.2*	28172631–28176106	PHT81228.1	PREDICTED: probable LRR receptor-like serine/threonine-protein kinase
P5	GWAS-*QTL5.2*	28974813–28975852	PHT81231.1	SAR8.2 precursor
P5	*QTL5.1*	18707644–18708354	PHT81101.1	PREDICTED: probable LRR receptor-like serine/threonine-protein kinase
P5	*QTL5.1*	18810592–18813153	PHT81104.1	PREDICTED: disease resistance protein RPP13-like
P5	*QTL5.1*	18943734–18945083	PHT81107.1	PREDICTED: disease resistance protein RPH8A-like
P5	*QTL5.1*	19074847–19077759	PHT81108.1	PREDICTED: disease resistance protein RPP13-like
P5	*QTL5.1*	19094517–19097099	PHT81109.1	PREDICTED: disease resistance protein RPP13-like
P5	*QTL5.1*	19153252–19169266	PHT81110.1	PREDICTED: probable LRR receptor-like serine/threonine-protein kinase
P5	*QTL5.1*	19288855–19292090	PHT81111.1	PREDICTED: LRR receptor-like serine/threonine-protein kinase EFR
P5	*QTL5.1*	19292463–19293858	PHT81112.1	PREDICTED: probable LRR receptor-like serine/threonine-protein kinase
P5	*QTL5.1*	19294140–19297023	PHT81113.1	PREDICTED: probable LRR receptor-like serine/threonine-protein kinase
P5	*QTL5.1*	19344986–19347916	PHT81114.1	PREDICTED: probable LRR receptor-like serine/threonine-protein kinase
P5	*QTL5.3*	35411316–35411978	PHT81306.1	Kunitz trypsin inhibitor 2-like
P5	*QTL5.3*	36088454–36089143	PHT81317.1	putative late blight resistance protein homolog R1B-17
P5	*QTL5.3*	36205447–36219154	PHT81318.1	PREDICTED: putative late blight resistance protein homolog R1C-3

^a^Peptide id given in the NCBI genome data base.

**Figure 5:**
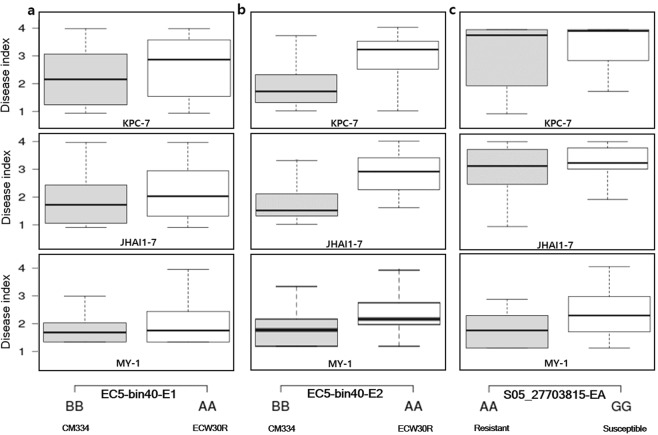
Box plots of tightly linked bins to QTLs in ECRILs and significantly associated GWAS-SNPs from chromosome P5. (**a**) ECRILs grouped based on the tightly linked bin to *QTL5.2* in environment E1. (**b**) ECRILs grouped based on the tightly linked bin to *QTL5.2* in environment E2. (**c**) Core collection GWAS (core collection) grouped based on the most significant SNP from QTL region 5.2.

We used the highly significant GWAS SNP (S05_27703815), with -log10(p) value of 11.23 (Supplementary Table S4), located close to the candidate genes to compare the resistance levels of the GWAS accessions. As shown in the box plots in Fig. 5c, the homozygous genotype (S05_27703815) AA was associated with increased resistance compared to the alternative homozygous genotype GG among the three PcRR isolates examined. We used the tightly linked bin marker EC5-bin40 of QTL segment *QTL5.2* overlapping with candidate genes to compare the resistance levels of the ECRILs. As shown in the box plots, the homozygous resistant genotype BB is associated with enhanced resistance compared to the homozygous susceptible genotype AA against all three isolates in both E1 and E2 (Fig. 5a,b).

The major QTL region *QTL5.1*, spanning 0.7 Mb, which was commonly detected against two isolates, JHAI1-7 and MY-1, contains 15 predicted genes (Supplementary Table S6), including genes associated with disease resistance, such as genes for six RLK domain-containing proteins and four NBS-LRR domain-containing proteins (Table 5). Finally, we used the tightly linked bin marker EC5-bin27 of QTL segment *QTL5.1* overlapping with candidate genes to compare the resistance levels of the ECRILs. As shown in the box plots, the homozygous resistant genotype BB is associated with enhanced resistance compared to the homozygous susceptible genotype AA against all three isolates in both E1 and E2 (Supplementary Fig. S6a,b).

Finally, *QTL5.3*, a QTL region against isolates KPC-7 and MY-1, was detected at 34.6–37 Mb on chromosome P5, with 28 predicted genes. Among these, three predicted genes are linked to disease resistance proteins, including two NBS-LRR domain-containing proteins and one Kunitz trypsin inhibitor 2-like protein (Table 5). We used the tightly linked bin marker EC5-bin51 of QTL segment *QTL5.3* overlapping with the candidate genes to compare the resistance levels of the ECRILs. As shown in the box plots, the homozygous resistant genotype BB is associated with enhanced resistance compared to homozygous susceptible genotype AA against all three isolates in both E1 and E2 (Supplementary Fig. S6c,d).

## Discussion

In this study, we combined traditional QTL mapping with GWAS to increase our understanding of PcRR resistance in pepper and detected common and race-specific QTLs for this trait. Although many QTL studies have been performed to investigate PcRR using biparental QTL mapping^4,9,10,20,21^, this is the first comprehensive study of PcRR resistance loci for multiple *P. capsici* isolates in multiple environments using biparental QTL mapping and GWAS.

### PcRR resistance QTLs

Due to the rapid genetic evolution and diversity of *Phytophthora*, there is an urgent need to breed pepper with resistance to multiple PcRR isolates^4,35^. Analysis of the differential responses of ECRILs and GWAS accessions against the three PcRR isolates provided us with valuable information about the range and frequency distribution of symptom development and virulence levels (Fig. 1). The mean disease scores of the resistant GWAS accessions were lower than those of the ECRILs (Supplementary Table S2), pointing to higher allelic diversity for PcRR resistance in the GWAS core collection. Furthermore, the high heritability of resistance (Table 1) points to the additive effects of genes, offering an excellent opportunity for improving this trait through selection.

Breeding for resistance to PcRR is quite challenging; even resistance incorporated into commercial pepper lines can be readily overcome by highly virulent *P. capsici* isolates^2^. CM334 is a well-known source of PcRR resistance. The inheritance of PcRR resistance in CM334 is complex^9,10^, and the genetic dissection of PcRR resistance would greatly facilitate the improvement of pepper cultivars. Therefore, in this study, we aimed to identify QTLs for different *P. capsici* isolates categorized based on virulence in two different environments. QTL mapping for PcRR resistance revealed significant QTLs, including three major-effect QTLs on chromosome P5 and several isolate-specific minor-effect QTLs on various chromosomes. Among the several QTLs identified for PcRR resistance, a major QTL on chromosome P5 has been consistently identified in several resistance sources irrespective of race, isolate, and level of virulence^4,10^. In agreement with this finding, two QTLs (*QTL5.1* and *QTL5.3*) specific to single isolates and one QTL (*QTL5.2*) common to all three isolates of *P. capsici* were consistently detected on chromosome P5 in two environments. The location of *QTL5.2* detected in this study coincides with that of the previously identified locus *Pc5.1*^4,10,21^. The dominant resistance locus *CaPhyto*, conferring race-specific resistance to PcRR, was previously mapped to a genetic interval near *Pc5.1* in a 3.3-cM region between two SSR markers, *ZL6726* and *ZL6970*, in *C*. *annuum* accession PI201234.

Several minor-effect QTLs have previously been reported on various chromosomes^4,11,18–20,36^. The number of QTLs conferring PcRR resistance and their positions have varied among studies depending on the mapping populations used, disease screening methods, inoculum density, and isolate aggressiveness. In this study, we detected some minor QTLs with isolate specificity on chromosome P2 (*Kpc1* for isolate KPC-7) and chromosome P8 (*Jha8.1*, *Jha8.2*, *Jha11.1*, and *Jha11.2* for isolate JHAI1-7) and with no isolate specificity on chromosome P11 (Table 4). However, we could not compare the exact physical positions of these QTLs with previously detected QTLs due to the limited number of common markers and the lack of sequence information; instead, we used the closest markers for analysis.

In addition, epistatic interactions between PcRR resistance QTLs have been reported^11,36^. Consistent with these reports, we detected epistatic QTL interactions between resistance QTLs on chromosomes P5 and P11 for isolate KPC-7, on chromosomes P5 and P8 and chromosomes P5 and P11 for isolate JHAI1-7, and on chromosomes P5 and P11 for isolate MY-1 in both environments.

### Validation of PcRR loci via GWAS

We took advantage of biparental QTL mapping together with GWAS to identify new PcRR resistance-related loci and to validate known QTL regions. This approach proved to be effective for the identification of PcRR resistance loci. We identified 117 significant SNPs related to PcRR isolate KPC-7 across the chromosomes. However, only SNPs identified on chromosomes P7, P5, and P11 colocalized with QTLs detected in this study (Fig. 4), perhaps because the ECRILs included only two parental alleles compared to the highly diverse GWAS accessions^33^. Thus, numerous biparental populations developed using different resistant genetic resources were likely captured among all resistant loci.

GWAS is a high-resolution, powerful technique, as it captures chronological recombination events that have accumulated inside an association panel. PcRR resistance loci have previously been mapped onto chromosomes P1, P3, P4, P6, P8, P9, P10, P11, and P12^4,11,18–20,36^. To determine whether the GWAS-SNPs identified in this study are novel PcRR resistance SNPs, we compared their physical positions with the positions of previously reported QTLs. The GWAS-SNPs on chromosome P5 (at 27.0–29.5 Mb) coincided with major QTLs detected in previous studies^4,10,21^. Two GWAS-SNP peaks on chromosome P10 at 14.2 and 196 Mb colocalized with the previously detected QTL *Ph051-10-2* (*RAUDPC*) and the race-specific locus *PhR10*, respectively^17,19^. However, due to the limited availability of marker sequences, it was not possible to compare and validate all GWAS-SNPs with previously reported resistance loci linked to PcRR. The GWAS-SNPs associated with PcRR identified in this study could serve as novel resistance loci that could be used to incorporate resistance into ongoing pepper-breeding programs.

### Potential candidate genes for PcRR resistance

In this study, we identified several classes of genes associated with disease resistance in various QTL regions. A cluster of NBS-LRR domain-containing *R* genes was identified on chromosome P5: four NBS-LRR genes from *QTL5.1* (18.7–19.5 Mb), two from *QTL5.2* (27.3–29.2 Mb), and two from *QTL5.3* (34.6–37 Mb). These candidate genes share high sequence identity with *RPP13*-like NBS-LRR protein genes and represent potential candidates for PcRR resistance in pepper. *RPP13* is a CC (coiled-coil)-NBS-LRR domain-containing *R* gene that confers resistance to the oomycete pathogen *Peronospora parasitica* in *Arabidopsis thaliana*^37^.

Plant genomes generally contain numerous NBS-LRR class *R* genes, which are often clustered on specific chromosomes due to tandem and segmental duplications^38–41^. Several dominant *R* genes, such as *R1* from potato^42^, *Ph*-*3* from tomato^43^, and *RpsUN1*, *RpsUN2*, and *Rpg1-b* from soybean^44,45^, belong to NBS-LRR domain-containing complex *R* gene clusters. *R* gene clusters could function as hotspots for the generation of novel *R* genes and enhance the possibility of structural and copy number variation through various DNA recombination and rearrangement mechanisms, including duplications, unequal crossing over, gene conversion, and diversifying selection, which could account for the gain or loss of resistance^45–47^. The candidate *R* gene clusters identified in this study could account for enhanced durability and resistance to PcRR in pepper. In addition to the NBS-LRR *R* gene clusters, we identified six RLKs from *QTL5.1* (18.7–19.5 Mb) and three from *QTL5.2* (27.3–29.2 Mb) on chromosome P5. RLKs are extracellular surface (also called pattern recognition) receptors that play crucial roles in plant defense-related processes, including both host and non-host defense responses^48^. Therefore, the RLK genes identified in this study represent strong candidates for PcRR resistance genes.

We also identified *SAR8.2* in *QTL5.2* (27.3–29.2 Mb), a systemic acquired resistance (SAR)-related gene known as *CASAR82A*. *CASAR82A* localizes to the phloem and epidermal cells of pepper leaf and stem tissues infected by *Colletotrichum coccodes* and *P. capsici* or treated with salicylic acid^48^. The involvement of the pepper *SAR8.2* gene in pathogen infection and environmental stress responses and its use as a marker of abiotic elicitors^49^ suggests it might also be an important candidate gene for PcRR resistance.

Kunitz trypsin inhibitor 2-like protein is involved in programmed cell death (PCD) in Arabidopsis. Arabidopsis serine protease (Kunitz trypsin) inhibitor (KTI1) appears to be a functional KTI when induced in bacteria and *in planta*. *Atkti1* expression is induced at a late stage of infection in response to fungal and bacterial elicitors and to salicylic acid. RNAi silencing of *AtKTI1* increased the rate of lesion development in leaf tissue after infiltration with the PCD-eliciting fungal toxin fumonisin B1 (FB1) or the avirulent bacterial pathogen *Pseudomonas syringae* pv. tomato DC3000 carrying avrB (Pst avrB)^50^. In this study, one gene encoding a Kunitz trypsin inhibitor 2-like protein was identified in a major QTL region on chromosome P5, *QTL5.3* (34.6–37 Mb), suggesting it is a candidate gene for PcRR. However, to uncover the roles of intra- and extracellular immune receptors in disease resistance, further analyses of the candidate genes should be performed, including targeted knockdown of candidate genes and subsequent molecular analyses.

In this study, we combined the use of biparental QTL mapping and GWAS to identify QTLs and candidate genes for PcRR resistance in pepper. Based on the genetic map and phenotypic data, 14 significant QTLs were identified with R^2^ values ranging from 3.73 to 38.99% among the three PcRR isolates in their respective environments. Two of these QTLs on chromosome P5 showed major effects, with a combined R^2^ > 60%. GWAS identified 117 highly significant SNPs associated with PcRR across the genome for isolates KPC-7 and JHAI1-7. The additional loci detected through GWAS might enhance the accuracy of selection for PcRR resistance. The use of SNP markers associated with the candidate genes or QTLs could accelerate MAS and genomic selection in pepper breeding programs for PcRR resistance. The predicted candidate genes for PcRR resistance lay the foundation for the molecular dissection of PcRR resistance.

## Materials and Methods

### Plant materials and DNA extraction

The ECRIL mapping population comprised 188 F_7:8_ ECRILs developed from a cross between resistant *C*. *annuum* line CM334 and susceptible line ECW30R. The single seed descent method was used for ECRIL development, with shuttle breeding. The population was abbreviated as “EC”, based upon the first letter of parental accessions. The plants were grown in plastic pots in a greenhouse. GWAS was carried out on a core collection comprising 352 *Capsicum* accessions^32^. Both parents of the ECRIL population were included in the core collection. Genomic DNA was extracted from young leaf tissues of plants of the ECRILs and the association population at the seedling stage using the CTAB (cetyl trimethyl ammonium bromide) method as described previously^51^.

### Disease assay and resistance scoring

Resistance against PcRR in the ECRILs was evaluated under two different environments: the Seoul National University (SNU) farm at Suwon, Republic of Korea (2017) (E1: Environment 1) and the Korea Research Institute of Chemical Technology (KRICT), Daejeon, Republic of Korea (2018) (E2: Environment 2). The GWAS core collection (consisting of 352 *Capsicum* accessions) was also evaluated in two different environments. Resistance screening was carried out in 2017 with isolate KPC-7 at Rural Development Authority (RDA), Jeonju, Republic of Korea (EA: Environment A), while isolates JHAI1-7 and MY-1 were utilized in 2018 at SNU (E1: Environment 1). *P. capsici* isolates MY-1, JHAI1-7, and KPC-7 (Supplementary Table S1) used in this study were kindly provided by Dr. Choi (KRICT). Isolates MY-1, JHAI1-7, and KPC-7 were reported to have low, moderate, and high levels of virulence, respectively^10,52^. To prepare the inoculum, the *P. capsici* isolates were cultured on V8-agar medium and incubated at 27 °C for mycelium growth for five days. The plates were flooded with distilled water to harvest the mycelia. The spore concentration was quantified using a hemocytometer, and the spore suspension density was adjusted to approximately 5 × 10^4^ sporangia per mL prior to inoculation. The ECRILs (188 F_7:8_ plants) and GWAS panel of 352 core collection accessions were scored for disease resistance with 10 replications per line. The plants were grown in 50-cell plastic trays, and inoculation was carried out by drenching plants at the 4–6 true leaf stage with 5 mL of spore suspension at the base of the stem using a dispenser. The inoculated plants were maintained under protected conditions at 24–30 °C and frequently watered to facilitate disease establishment. *C*. *annuum* cultivars CM334, PI201234, and Mohanjilju (commercial F_1_ hybrid, Syngenta Korea) were used as the resistant controls, whereas *C*. *annuum* cultivars ECW30R, Tean, and Geumsugangsan (commercial F_1_ hybrid, Takii Korea) were used as the susceptible controls to compare the disease resistance levels. The disease indexes were scored at three weeks post inoculation based on a previously described disease scale of 1–4, where 1 = no visible symptoms, 2 = dark lesion visible at the base of the stem but surviving without wilting, 3 = wilting with a dark lesion at the base of the stem, and 4 = wilting and death of the whole plant^34,53^.

### Genotyping-by-sequencing

Genotyping-by-sequencing was performed for the ECRILs and GWAS core collection as described previously^23^. Briefly, genomic DNA was diluted, adjusted to a concentration of 50 ng/µL per ECRIL, and digested with the restriction enzymes *Eco*RI and *Mse*I using a SBG 100‐Kit v2.0 (Keygene N.V., Wageningen, the Netherlands). For the GWAS core collection population, GBS libraries were constructed manually using restriction enzymes *Pst*I/*Mse*I and *EcoR*I/*Mse*I as described previously^23,54^. Following the ligation of selective adaptors, the libraries were amplified using selective primers containing “GA” for the ECRILs and “TA” for the GWAS core collection population. Amplified libraries consisting of 188 ECRILs and two replicates of susceptible parents were pooled into a single tube. Libraries consisting of the GWAS core collection populations were pooled into five tubes. The pooled libraries were sequenced on the Illumina HiSeq. 2000 platform at Macrogen (Macrogen, Inc. Seoul, Korea). Trimming and quality control of the GBS raw data were performed using a CLC Genomics Workbench v6.5 (Qiagen, Aarhus, Denmark) with a minimum read length of 80 bp and a minimum quality score of Q20. Filtered reads were aligned to the *C*. *annuum* cv. CM334 reference genome (chromosome v1.6, http://peppergenome.snu.ac.kr) using the Burrows-Wheeler Aligner (BWA)^55^. Filtering and SNP calling were performed using the Genome Analysis Toolkit (GATK) Unified Genotyper version 3.3-0. SNPs in the ECRILs were filtered with QUAL value > 20 and minimum read depth of 3. SNPs in the GWAS core collection population were filtered with minor allele frequency (MAF) > 0.03, calling rate >0.6, and inbreeding coefficient (F) > 0.8.

### Construction of bin map and QTL analysis

SNPs with unequal segregation and > 10% missing data were excluded from genetic linkage map construction. For genetic linkage map construction, bins were treated as genetic markers. The sliding window approach was used to impute the missing data and to detect recombination breakpoints as described previously^23^. To assign genetic positions to the bins, arranged bins were mapped with a LOD (logarithm of the odds) threshold of 3.0 and a distance threshold of 30 cM using CarthaGene software. The Kosambi mapping function was used to infer genetic distances between markers in centimorgans (cM). QTL analysis was performed using Composite Interval Mapping (CIM) with Windows QTL Cartographer 2.5^56^. The 1000-permutation test (*P* < 0.05) was performed to determine the LOD threshold for significance of each QTL. Explanations of the percentage of phenotypic variance [R^2^ (%)] and additive effects for each QTL were obtained using the same software. Epistatic effects and the interactions of major QTLs were examined using the Multiple Interval Mapping (MIM) option under the command “Scan through QTL mapping results file” and refined further through “Testing for existing QTLs” with a Bayesian Information Criterion (BIC-X) model using default options. The physical locations of the QTLs were compared with the genetic locations of bins linked to the significant QTLs. The *C*. *annuum* cv. CM334 reference genome (chromosome v1.6, http://peppergenome.snu.ac.kr) was used to compare the positions of significant SNPs in the QTLs and GWAS core collection identified in this study and with previously reported linked markers. The marker sequences of previous studies were retrieved using https://solgenomics.net/search/markers and BLAST analysis of the reference genomes. If the sequence information for a particular linked marker was not publically accessible, the closest marker was used.

### Genome-wide association analyses for PcRR resistance

The 507,713 filtered SNPs discovered from the 352 core collection population were used for GWAS mapping. Population structure (PCA and kinship matrixes) and genome-wide association (GWAS) were estimated with the Compressed Mixed Linear Model (CMLM) using the R package Genomic Association and Prediction Integrated Tool (GAPIT)^57^ with default parameters. All probabilities generated in the association runs were transformed by -log_10_ P (FDR *p*-value < 0.05). Scores for an individual chromosome were then inspected in Manhattan plots to determine whether the SNPs reached the significance threshold. The -log_10_ *p-*values of SNPs from GWAS were adjusted by^58^ multiple test correction, and the adjusted cutoff value for accepting thresholds was set to -log_10_ (P) value ≥ 7.0. The haplotype blocks of the GWAS core collection were estimated using PLINK v1.9 with the following settings:–no-parents–allow-no-sex–blocks-max-kb 2000–blocks-inform-frac 0.9–blocks-strong-highci 0.85–blocks-recomb-highci 0.8^59^. Haplotype block analysis for the chromosomes identified with significant SNPs was performed using Haploview 4.2 software^60^. The significant SNP data sets from GAPIT were used to calculate pair-wise LD between SNPs. A 1% cutoff was used. Haplotype blocks in the region were defined with the confidence interval method^61^.

### Identification of candidate genes

To identify candidate genes associated with PcRR resistance, the positions of highly significant and linked bin markers within QTLs on the genetic map were compared with their physical positions on the pepper reference genome (v1.6), and 1-Mb upstream and downstream sequences were mined for genes associated with PcRR disease resistance. Similarly, 1-Mb sequence information for the highly significant SNPs associated with the PcRR resistance trait from the GWAS peak was used for BLAST (blastn) analysis against the *C*. *annuum* cv. CM334 reference genome database (chromosome v1.6, http://peppergenome.snu.ac.kr) to identify candidate PcRR resistance genes.

### Statistical analysis to estimate genetic variability against PcRR

The mean disease scores of ten plants per ECRIL and GWAS core collection accessions were used to calculate the frequency distribution, coefficient of variation (CV%), range, and genetic variability parameters for three different isolates in their respective environments. Broad-sense heritability (H^2^_bs_) was calculated with the formula (suggested previously^62^) ${H}^{2}=\frac{{\hat{\sigma }}_{{\rm{g}}}^{2}}{{\hat{\sigma }}_{p}^{2}}\times 100$, where, ${\hat{\sigma }}_{{\rm{g}}}^{2}$ is the genetic variance and ${\hat{\sigma }}_{p}^{2}$ is the phenotypic variance. The Pearson correlation was calculated using Rstudio.

## Supplementary information

Identifying candidate genes for Phytophthora capsici resistance in pepper (Capsicum annuum) via genotyping-by-sequencing-based QTL mapping and genome-wide association study

Supplementary Table S6.
