# Supplementary material for: Identifying candidate genes for Phytophthora capsici resistance in pepper (Capsicum annuum) via genotyping-by-sequencing-based QTL mapping and genome-wide association study
Source: Sci Rep. 2019 Jul 10;9:9962. doi: 10.1038/s41598-019-46342-1 (PMC6620314; doi:10.1038/s41598-019-46342-1)
Supplement: Supplementary file 1 — Identifying candidate genes for Phytophthora capsici resistance in pepper (Capsicum annuum) via genotyping-by-sequencing-based QTL mapping and genome-wide association study [file 41598_2019_46342_MOESM1_ESM.pdf]

Supplementary Material

Identifying candidate genes for *Phytophthora capsici* resistance in pepper (*Capsicum annuum*) via genotyping-by-sequencing-based QTL mapping and genome-wide association study

Muhammad Irfan Siddique[1], Hea-Young Lee[1], Na-Young Ro[2], Koeun Han[1], Jelli Venkatesh[1], Solomon Abate Mekonnen[1], Abhinandan Surgonda Patil[1], Amornrat Changkwian[1] Jin-Kyung Kwon[1], Byoung-Cheorl Kang[1]*

[*]**Corresponding author:** Byoung-Cheorl Kang

E-mail: bk54@snu.ac.kr

1. Supplemental files

Supplementary Figure and Table legends

Supplementary Figure S1. Disease scale (1 to 4) used to distinguish the resistant and susceptible genotypes, where 1 = no visible symptoms, 2 = dark lesion visible on the base of the stem but surviving without wilting, 3 = wilting, with a dark lesion at the base of the stem, and 4 = wilting and death of the whole plant.

Supplementary Figure S2. Pearson correlation matrix of PcRR disease index for ECRILs in two environments (E1 and E2).

Supplementary Figure S3. Pearson correlation matrix of PcRR disease index for the GWAS (core collection) in two environments (EA and E1).

Supplementary Figure S4. High-density genetic linkage map of ECRILs comprising 2,663 bins covering a genetic distance of 1,428 cM based on genotyping-by-sequencing.

Supplementary Figure S5. Manhattan plots based on GBS-GWAS for PcRR resistance. (a) Significant SNPs associated with PcRR isolate JHAI1-7 (b) against low-virulent isolate MY-1.

Supplementary Figure S6. Box plots of the tightly linked bin to PcRR resistance QTLs and GWAS-SNPs associated with PcRR resistance against three isolates. (a and b) ECRILs grouped based on the tightly linked bin to QTL 5.1 in environments E1 and E2. (c and d) ECRILs grouped based on the tightly linked bin to QTL 5.3 in environments E1 and E2. (e) Core collection population grouped based on the most significant SNP on the lower arm of chromosome P5.

Supplementary Table S1. Details about the three *P. capsici* isolates used in this study to screen the ECRILs and GWAS core collection

Supplementary Table S2. Details about the resistant accessions from the GWAS core collection.

Supplementary Table S3. Epistatic effects of major QTLs in ECRILs against three PcRR isolates evaluated in two environments

Supplementary Table S4. Significant SNPs associated above the threshold $-\log_{10}$ *p*-values for PcRR isolates KPC-7 and JHAI1-7 (bold font) in the GWAS core collection.

Supplementary Table S5. Haplotype blocks estimated by genotyping-by-sequencing of the GWAS core collection.

Supplementary Table S6. Candidate genes located in the vicinity of the GWAS-SNPs and biparental QTLs and their functional annotations.

Attached as an excel file

Supplementary Figure S1

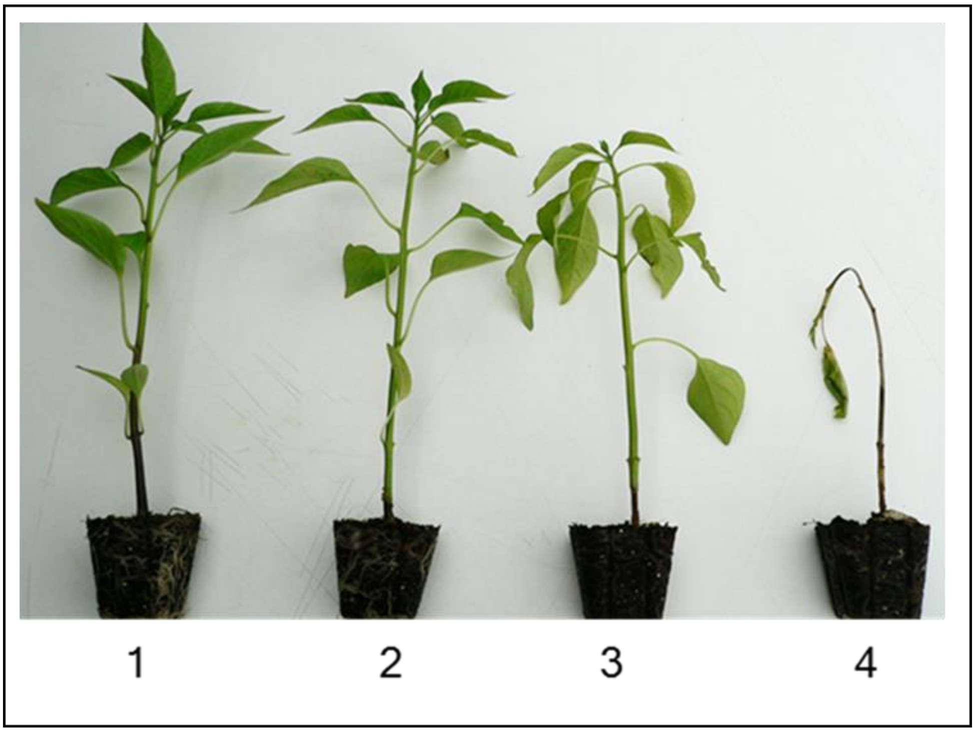

Supplementary Figure S1. Disease scale (1 to 4) used to distinguish the resistant and susceptible genotypes, where 1 = no visible symptoms, 2 = dark lesion visible on the base of the stem but surviving without wilting, 3 = wilting, with a dark lesion at the base of the stem, and 4 = wilting and death of the whole plant.

Supplementary Figure S2

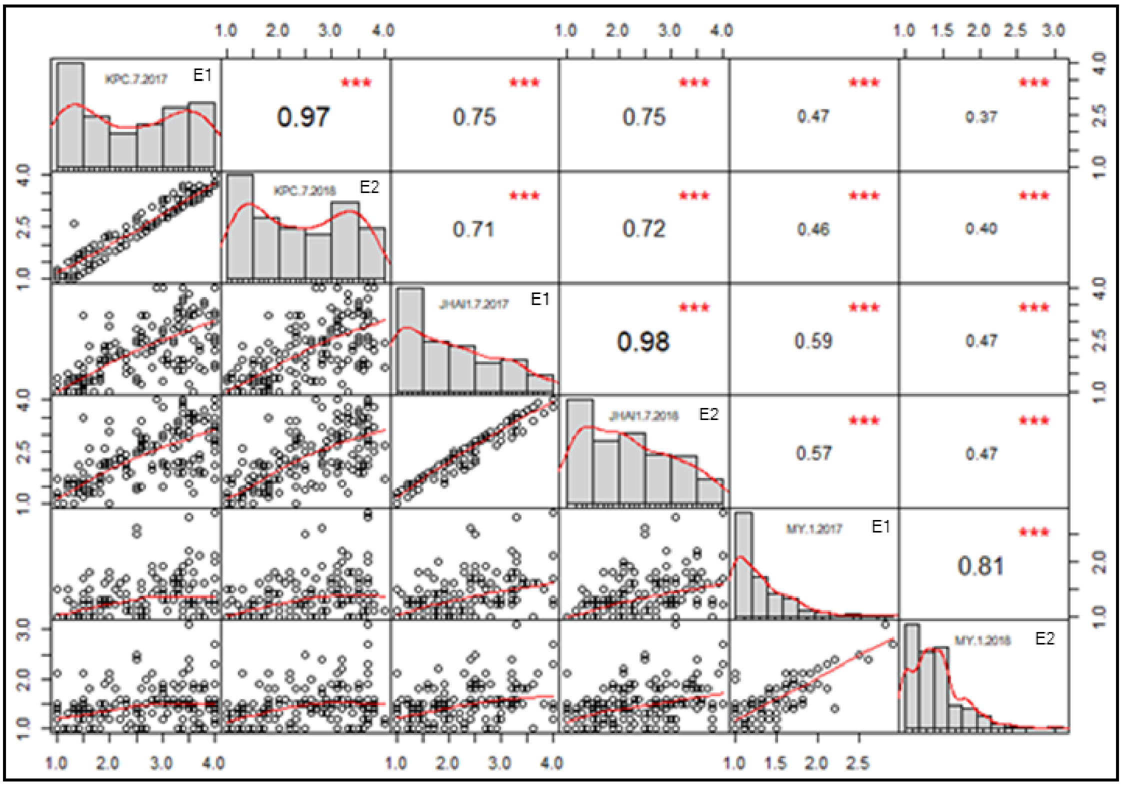

Supplementary Figure S2. Pearson correlation matrix of PcRR disease index for ECRILs in two environments (E1 and E2).

Supplementary Figure S3

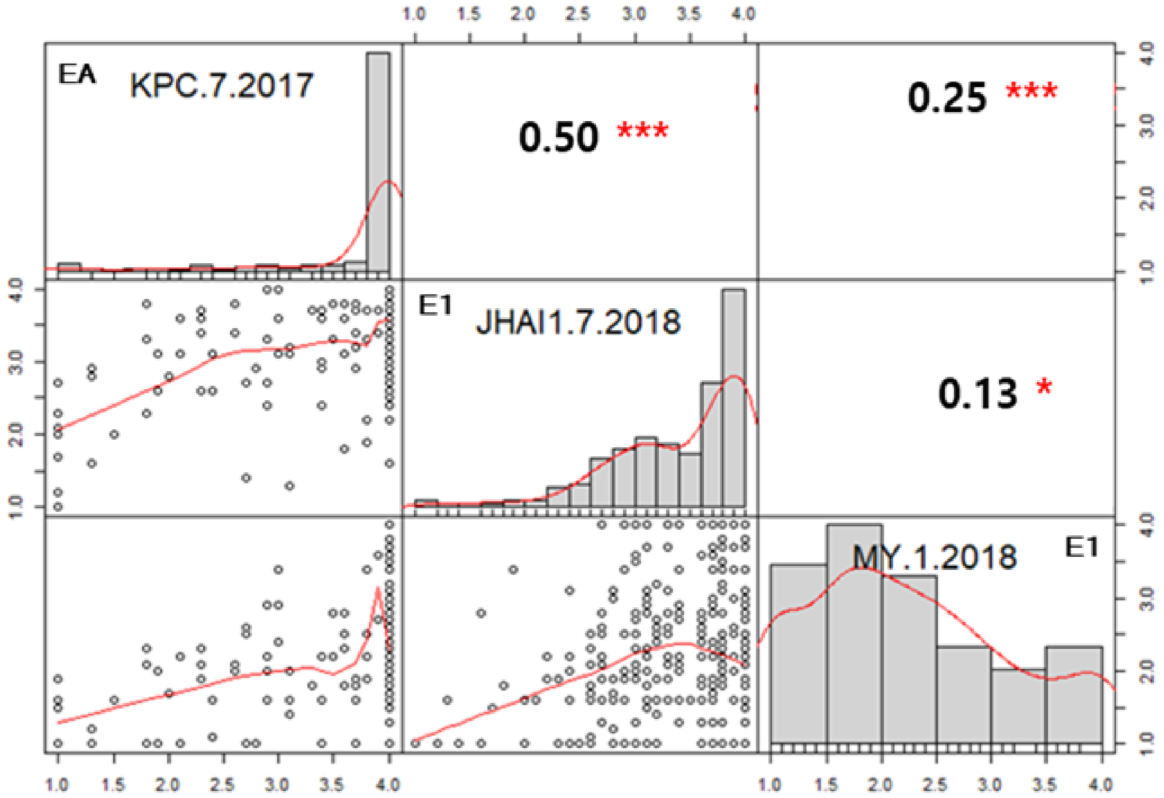

Supplementary Figure S3. Pearson correlation matrix of PcRR disease index for the GWAS (core collection) in two environments (EA and E1).

Supplementary Figure S4

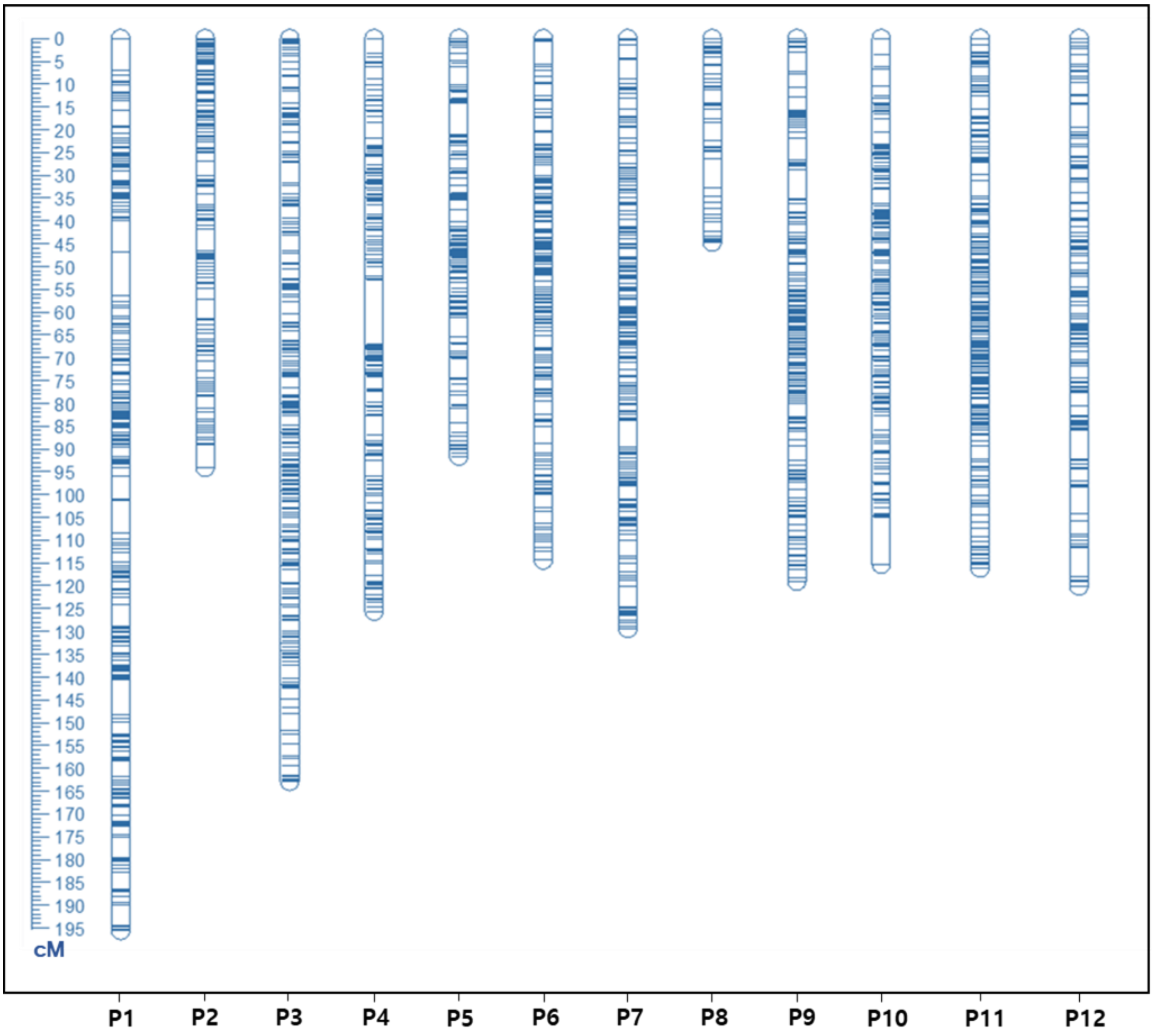

Supplementary Figure S4. High-density genetic linkage map of ECRILs comprising 2,663 bins covering a genetic distance of 1,428 cM based on genotyping-by-sequencing.

Supplementary Figure S5

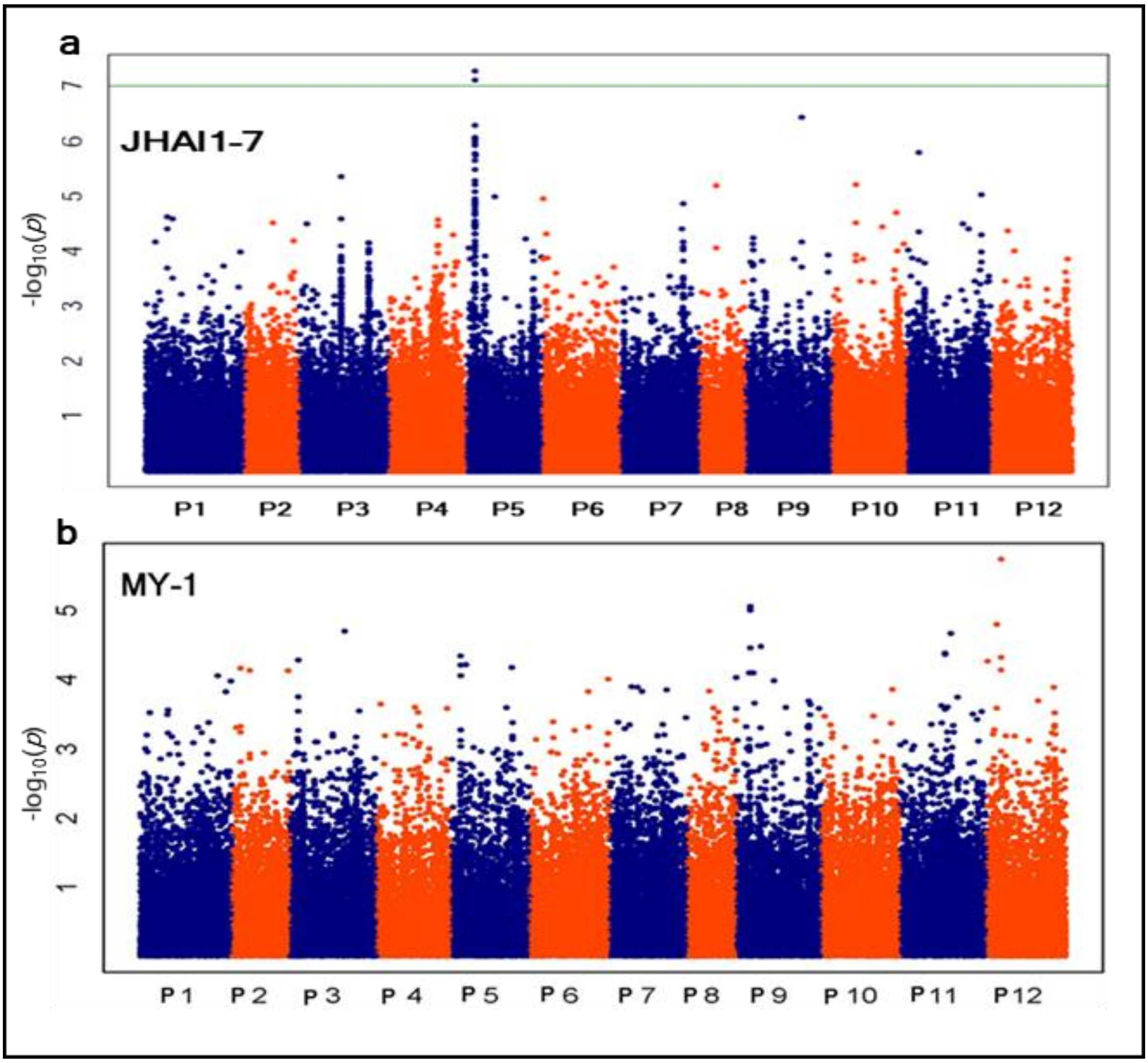

Supplementary Figure S5. Manhattan plots based on GBS-GWAS for PcRR resistance. (a) Significant SNPs associated with PcRR isolate JHAI1-7 (b) against low-virulent isolate MY-1.

Supplementary Figure S6

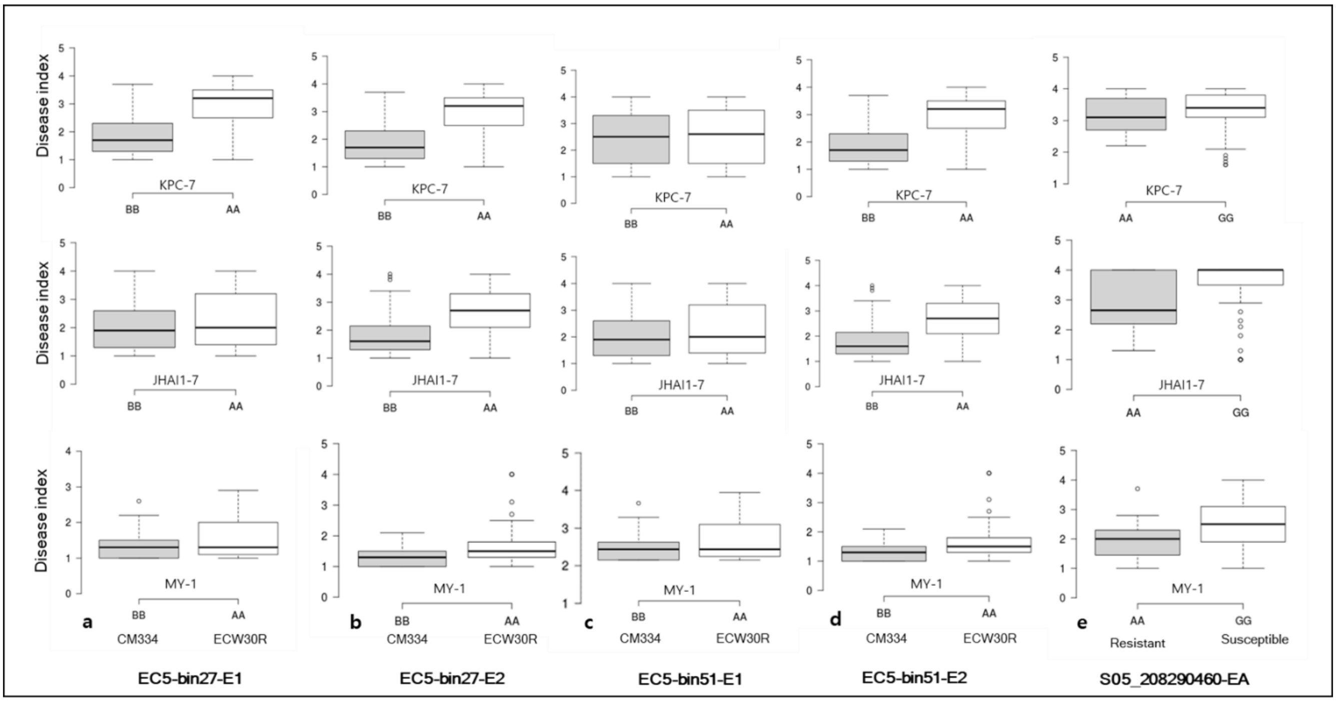

Supplementary Figure S6. Box plots of the tightly linked bin to PcRR resistance QTLs and GWAS-SNPs associated with PcRR resistance against three isolates. (a and b) ECRILs grouped based on the tightly linked bin to QTL 5.1 in environments E1 and E2. (c and d) ECRILs grouped based on the tightly linked bin to QTL 5.3 in environments E1 and E2. (e) Core collection population grouped based on the most significant SNP on the lower arm of chromosome P5.

Supplementary Table S1. Details about the three *P. capsici* isolates used in this study to screen the ECRILs and GWAS core collection.

Isolate	Collection site	Host	Virulence	Mating type	Source	References
KPC-7	Kangwon/Korea	*C. annuum*	High	A1	KRIPT	Jo et al. (2014)
JHAI1-7	Chungbuk/Korea	*C. annuum*	Moderate	A1	KRIPT	Liu et al. (2014)
MY-1	Kangwon/Korea	*C. annuum*	Low	A2	KRIPT	Jo et al. (2014)

Supplementary Table S2. Details about the resistant accessions from the GWAS core collection.

**Accession name**	**IT number**[X]	**Species**	**Origin**	**Resistance score**[Y]		
				**MY-1**	**KPC-7**	**JHAI1-7**
Szechwan 8	IT 158280	*C. annuum*	Taiwan	1	1.8	1
Pangalengan-1	IT 158714	*C. annuum*	Indonesia	1	1.7	1
Ikdosan	IT 236666	*C. annuum*	China	1	1.9	1
Jungangjongmyo-2000-5032	IT 236424	*C. annuum*	-	1	2	1
Ibam	IT 231151	*C. annuum*	-	1	1.2	1
CM334	IT 250209	*C. annuum*	Mexico	1	1	1
CM331	IT 236422	*C. annuum*	Mexico	1	1	1
PI 439449	IT 264121	*C. chinense*	Peru	1	2	1
AC2212	IT 294726	*C. chinense*	-	1	1.2	1
YCM334	K150013	*C. annuum*	-	1	1.5	1.6
PI 201234	IT 294726	*C. annuum*	-	1	1.7	1.3

[X] GenBank accession number.

[Y] Disease index.

Supplementary Table S3. Epistatic effects of major QTLs in ECRILs against three PcRR isolates evaluated in two environments.

Isolate	Environment*year	QTLs and their epistatic interactions	R^2 (%)	Total R^2 (%)
KPC-7	E1-SNU*2017	*QTL5.2*	24.4	42
		QTL5.3	15.1	
		QTL5.2 x *QTL5.3*	2.5	
		QTL5.2	24.4	41.1
		E1Kpc-11.1	5.5	
		QTL5.2 x *E1Kpc-11.1*	11.2	
	E2-KRICT*2018	*QTL5.2*	25.6	39.3
		QTL5.3	7.5	
		QTL5.2 x *QTL5.3*	6.2	
JHAI1-7	E1-SNU*2017	*QTL5.2*	28.2	35.1
		Jha8.1	4.5	
		QTL5.2 x *Jha8.1*	2.4	
		QTL5.2	28.2	44
		Jha11.2	13.5	
		QTL5.2 x *Jha11.2*	2.3	
	E2-KRICT*2018	*QTL5.2*	32.5	43
		Jha11.1	8.4	
		QTL5.2 x *Jha11.1*	2.1	
MY-1	E1-SNU*2017	*QTL5.2*	8.7	17.4
		My11	4.8	
		QTL5.2 x *My11*	3.9	
	E2-KRICT*2018	*QTL5.2*	11.6	16.6
		My11	2.7	
		QTL5.2 x *My11*	2.3	

Supplementary Table S4. Significant SNPs associated above the threshold -log$_{10}$ *p*-values for PcRR isolates KPC-7 and JHAI1-7 (bold font) in the GWAS core collection.

SNP-ID	Chr.	Position (bp)	Alleles	*p*-value	-log10(p)	FDR_*p*-value	MAF	R^2 with SNP	Effect Est.
S02_149068682	P2	149,068,682	G/A	8.13E-09	8.09	8.77E-05	0.0430	0.2332	0.5525
S03_5274098	P3	5,274,098	G/A	4.01E-08	7.40	2.43E-04	0.0415	0.2244	0.5288
S05_27703815	P5	27,703,815	G/A	5.84E-12	11.23	7.41E-07	0.0920	0.2745	0.4714
S05_27761792	P5	27,761,792	C/T	8.67E-10	9.06	1.69E-05	0.0623	0.2457	-0.4830
S05_27807397	P5	27,807,397	T/G	4.97E-09	8.30	5.87E-05	0.3635	0.2359	0.3283
S05_27816851	P5	27,816,851	T/C	1.16E-09	8.93	2.11E-05	0.0890	0.2441	0.4075
S05_27816914	P5	27,816,914	T/C	1.45E-11	10.84	9.20E-07	0.0742	0.2692	0.4948
S05_27816918	P5	27,816,918	G/A	1.45E-11	10.84	9.20E-07	0.0742	0.2692	0.4948
S05_27879870	P5	27,879,870	A/T	1.05E-12	11.98	2.66E-07	0.0682	0.2846	-0.5507
S05_27950402	P5	27,950,402	A/G	2.49E-08	7.60	1.81E-04	0.3220	0.2270	-0.2961
S05_27950407	P5	27,950,407	A/G	8.81E-09	8.06	8.77E-05	0.3650	0.2327	-0.3247
S05_27950442	P5	27,950,442	T/C	8.81E-09	8.06	8.77E-05	0.3650	0.2327	0.3247
S05_27950452	P5	27,950,452	A/T	7.43E-09	8.13	8.20E-05	0.3591	0.2337	-0.3236
S05_27950453	P5	27,950,453	G/A	2.92E-10	9.54	9.25E-06	0.2582	0.2519	0.4017
S05_28046041	P5	28,046,041	G/A	1.56E-10	9.81	5.67E-06	0.1172	0.2555	0.4176
S05_28046047	P5	28,046,047	A/G	9.78E-09	8.01	9.19E-05	0.2745	0.2322	-0.3710
S05_28046048	P5	28,046,048	G/A	4.81E-12	11.32	7.41E-07	0.0816	0.2756	0.4878
S05_28122588	P5	28,122,588	A/T	2.18E-09	8.66	3.35E-05	0.0593	0.2405	-0.4749
S05_28122629	P5	28,122,629	G/A	2.18E-09	8.66	3.35E-05	0.0593	0.2405	0.4749
S05_28123055	P5	28,123,055	G/A	1.27E-09	8.90	2.22E-05	0.2211	0.2436	0.3980
S05_28129446	P5	28,129,446	C/T	1.41E-11	10.85	9.20E-07	0.0786	0.2694	-0.4893
S05_28129512	P5	28,129,512	T/C	8.18E-10	9.09	1.66E-05	0.3739	0.2461	0.3859
S05_28134305	P5	28,134,305	G/A	4.72E-10	9.33	1.09E-05	0.0772	0.2492	0.4413
S05_28134313	P5	28,134,313	C/T	7.10E-09	8.15	8.01E-05	0.0742	0.2339	-0.4136
S05_28134344	P5	28,134,344	C/T	4.72E-10	9.33	1.09E-05	0.0772	0.2492	-0.4413
S05_28134355	P5	28,134,355	C/T	4.72E-10	9.33	1.09E-05	0.0772	0.2492	-0.4413
S05_28134380	P5	28,134,380	T/C	9.72E-09	8.01	9.19E-05	0.1157	0.2322	0.3691
S05_28136336	P5	28,136,336	T/C	4.32E-10	9.36	1.09E-05	0.0653	0.2497	0.4770
S05_28136347	P5	28,136,347	G/A	4.32E-10	9.36	1.09E-05	0.0653	0.2497	0.4770
S05_28163580	P5	28,163,580	G/A	1.06E-11	10.98	9.20E-07	0.0712	0.2710	0.5088
S05_28163627	P5	28,163,627	G/A	4.78E-11	10.32	2.21E-06	0.0757	0.2623	0.4852
S05_28225171	P5	28,225,171	A/G	4.32E-08	7.36	2.52E-04	0.2389	0.2240	-0.3286
S05_28225177	P5	28,225,177	A/G	2.53E-09	8.60	3.56E-05	0.1276	0.2397	-0.4253
S05_28225178	P5	28,225,178	C/T	5.50E-11	10.26	2.32E-06	0.0712	0.2615	-0.4874
**S05_28225198**	**P5**	***28,225,198**	**A/G**	**8.62E-13**	**12.06**	**2.66E-07**	**0.0653**	**0.2858**	**-0.5642**
S05_28261683	P5	28,261,683	G/C	9.74E-10	9.01	1.83E-05	0.1053	0.2451	0.4090
S05_28261689	P5	28,261,689	C/T	2.51E-11	10.60	1.28E-06	0.0727	0.2660	-0.4909
S05_28261695	P5	28,261,695	T/C	9.16E-11	10.04	3.58E-06	0.1261	0.2585	0.4061
S05_28261710	P5	28,261,710	C/T	2.51E-11	10.60	1.28E-06	0.0727	0.2660	-0.4909
S05_28274277	P5	28,274,277	C/G	1.97E-08	7.71	1.47E-04	0.0712	0.2283	-0.4071
S05_28393613	P5	28,393,613	C/T	1.69E-09	8.77	2.77E-05	0.0801	0.2420	-0.4358
S05_28524281	P5	28,524,281	T/C	7.99E-08	7.10	3.59E-04	0.3516	0.2206	0.3294
S05_28524343	P5	28,524,343	C/T	3.95E-09	8.40	5.42E-05	0.0549	0.2372	-0.4939
**S05_28634197**	**P5**	**28,634,197**	**G/A**	**5.65E-08**	**7.25**	**2.99E-04**	**0.0653**	**0.2225**	**0.4242**
S05_28670550	P5	28,670,550	T/C	7.50E-08	7.12	3.53E-04	0.2685	0.2209	0.3536
S05_28670630	P5	28,670,630	A/G	1.51E-08	7.82	1.28E-04	0.0935	0.2298	-0.3745
S05_28673591	P5	28,673,591	C/T	1.49E-09	8.83	2.52E-05	0.2018	0.2427	-0.3609
S05_28673596	P5	28,673,596	G/T	7.13E-10	9.15	1.51E-05	0.1944	0.2468	-0.4116
S05_28676908	P5	28,676,908	T/C	1.35E-08	7.87	1.20E-04	0.3828	0.2304	0.3400
S05_28676914	P5	28,676,914	G/T	1.12E-08	7.95	1.03E-04	0.3739	0.2314	-0.3364
S05_28676957	P5	28,676,957	T/C	9.34E-09	8.03	9.12E-05	0.3813	0.2324	0.3435
S05_28825990	P5	28,825,990	T/C	7.81E-08	7.11	3.57E-04	0.2255	0.2207	0.3100
S05_29768389	P5	29,768,389	G/A	1.84E-08	7.74	1.39E-04	0.0386	0.2287	0.5791
S05_206043102	P5	206,043,102	G/A	3.11E-08	7.51	2.02E-04	0.1261	0.2258	0.3289
S05_206565568	P5	206,565,568	C/T	5.48E-08	7.26	2.95E-04	0.1261	0.2227	-0.3204
S05_207549766	P5	207,549,766	G/A	6.50E-10	9.19	1.44E-05	0.1469	0.2474	0.3360
S05_208290460	P5	208,290,460	G/A	1.91E-10	9.72	6.48E-06	0.0608	0.2543	0.5171
S05_208664944	P5	208,664,944	A/G	3.47E-08	7.46	2.22E-04	0.0519	0.2252	-0.4819
S05_208711022	P5	208,711,022	A/G	2.70E-08	7.57	1.93E-04	0.0445	0.2266	-0.5330
S05_208711026	P5	208,711,026	A/C	2.85E-08	7.55	1.94E-04	0.0445	0.2263	-0.5324
S05_208711043	P5	208,711,043	G/A	1.81E-08	7.74	1.39E-04	0.0430	0.2288	0.5456
S05_208814172	P5	208,814,172	C/T	5.27E-08	7.28	2.91E-04	0.0504	0.2229	-0.4766
S05_208958802	P5	208,958,802	G/A	7.77E-08	7.11	3.57E-04	0.0460	0.2207	0.4919
S05_208963284	P5	208,963,284	G/A	4.47E-09	8.35	5.70E-05	0.0504	0.2365	0.5150
S05_210409729	P5	210,409,729	G/T	2.87E-08	7.54	1.94E-04	0.0371	0.2262	-0.5652
S05_212675435	P5	212,675,435	C/T	3.08E-08	7.51	2.02E-04	0.1306	0.2258	-0.3159
S06_171517874	P6	171,517,874	G/A	6.35E-08	7.20	3.22E-04	0.0712	0.2218	0.3978
S06_171572930	P6	171,572,930	G/A	6.01E-09	8.22	6.94E-05	0.0638	0.2349	0.4568
S07_25745130	P7	25,745,130	A/G	2.78E-08	7.56	1.94E-04	0.0356	0.2264	-0.5825
S07_90664059	P7	90,664,059	G/A	5.17E-08	7.29	2.91E-04	0.0950	0.2230	0.3758
S07_90774844	P7	90,774,844	G/A	5.52E-08	7.26	2.95E-04	0.1098	0.2226	0.3590
S07_90803701	P7	90,803,701	A/G	9.04E-08	7.04	3.98E-04	0.2478	0.2199	-0.3161
S07_90992151	P7	90,992,151	T/G	5.83E-08	7.23	3.02E-04	0.1083	0.2223	0.3601
S07_91181618	P7	91,181,618	A/T	3.50E-08	7.46	2.22E-04	0.0920	0.2251	-0.3890
S07_91530204	P7	91,530,204	G/T	7.32E-08	7.14	3.47E-04	0.1142	0.2211	-0.3594

S07_91628543	P7	91,628,543	A/G	4.71E-09	8.33	5.70E-05	0.0846	0.2362	-0.4223
S07_91631607	P7	91,631,607	C/A	4.93E-08	7.31	2.81E-04	0.1202	0.2232	0.3621
S07_92170698	P7	92,170,698	C/T	2.73E-08	7.56	1.93E-04	0.1083	0.2265	-0.3666
S07_92247467	P7	92,247,467	C/T	5.83E-08	7.23	3.02E-04	0.1128	0.2223	-0.3666
S07_92730273	P7	92,730,273	C/A	1.51E-08	7.82	1.28E-04	0.1202	0.2298	0.3683
S07_198979154	P7	198,979,154	A/G	6.19E-08	7.21	3.18E-04	0.1973	0.2220	-0.2889
S09_263032829	P9	263,032,829	G/A	1.73E-08	7.76	1.37E-04	0.0519	0.2290	0.4977
S10_8558399	P10	8,558,399	C/T	4.40E-08	7.36	2.54E-04	0.0519	0.2239	-0.4906
S10_8593630	P10	8,593,630	G/T	4.52E-09	8.35	5.70E-05	0.0608	0.2365	-0.4973
S10_8715796	P10	8,715,796	G/T	1.61E-08	7.79	1.32E-04	0.0593	0.2294	-0.4888
S10_8715862	P10	8,715,862	G/A	1.61E-08	7.79	1.32E-04	0.0593	0.2294	0.4888
S10_8767770	P10	8,767,770	C/T	1.70E-08	7.77	1.37E-04	0.0668	0.2291	-0.4688
S10_8851316	P10	8,851,316	A/G	7.00E-08	7.15	3.38E-04	0.0475	0.2213	-0.5002
S10_8851319	P10	8,851,319	C/T	7.00E-08	7.15	3.38E-04	0.0475	0.2213	-0.5002
S10_14299301	P10	14,299,301	G/A	8.90E-08	7.05	3.96E-04	0.1439	0.2200	0.2920
S10_70178306	P10	70,178,306	A/G	7.67E-08	7.12	3.57E-04	0.3887	0.2208	0.2894
S10_112433434	P10	112,433,434	T/G	1.15E-08	7.94	1.04E-04	0.3516	0.2313	-0.2897
S10_148308205	P10	148,308,205	C/G	3.97E-08	7.40	2.43E-04	0.2433	0.2244	0.2909
S10_148799589	P10	148,799,589	T/C	3.90E-08	7.41	2.43E-04	0.2507	0.2245	-0.2966
S10_148799665	P10	148,799,665	T/G	4.02E-08	7.40	2.43E-04	0.2493	0.2244	-0.2969
S10_196014162	P10	196,014,162	T/A	3.78E-10	9.42	1.09E-05	0.0430	0.2504	0.6209
S11_75334209	P11	75,334,209	C/A	8.67E-09	8.06	8.77E-05	0.1024	0.2328	0.3792
S11_75334214	P11	75,334,214	C/T	2.37E-08	7.62	1.75E-04	0.1068	0.2273	-0.3602
S11_75972407	P11	75,972,407	G/A	5.37E-08	7.27	2.93E-04	0.1053	0.2228	0.3609
S11_75972413	P11	75,972,413	A/T	4.37E-09	8.36	5.70E-05	0.0979	0.2367	-0.4041
S11_75972450	P11	75,972,450	G/A	6.76E-08	7.17	3.33E-04	0.1113	0.2215	0.3493
S11_90049314	P11	90,049,314	G/A	9.90E-08	7.00	4.29E-04	0.1113	0.2194	0.3446
S11_92434227	P11	92,434,227	C/T	9.09E-08	7.04	3.98E-04	0.1068	0.2199	-0.3533
S11_112878746	P11	112,878,746	G/A	8.62E-09	8.06	8.77E-05	0.0623	0.2329	0.4743
S12_34415370	P12	34,415,370	G/A	4.28E-08	7.37	2.52E-04	0.0623	0.2240	0.4499
S12_34415387	P12	34,415,387	C/A	7.25E-08	7.14	3.47E-04	0.0638	0.2211	0.4399
S12_34415405	P12	34,415,405	G/A	6.51E-08	7.19	3.24E-04	0.0445	0.2217	0.5157
S12_42543593	P12	42,543,593	A/G	6.40E-08	7.19	3.22E-04	0.3145	0.2218	0.2587
S12_57164515	P12	57,164,515	G/A	4.64E-09	8.33	5.70E-05	0.1365	0.2363	0.3546
S12_224191357	P12	224,191,357	A/T	7.95E-08	7.10	3.59E-04	0.0564	0.2206	-0.4523
S12_230507879	P12	230,507,879	C/A	2.34E-09	8.63	3.40E-05	0.0549	0.2401	0.5094
S12_230507903	P12	230,507,903	C/G	2.34E-09	8.63	3.40E-05	0.0549	0.2401	-0.5094
S12_230514706	P12	230,514,706	C/T	1.78E-08	7.75	1.39E-04	0.0593	0.2288	-0.4635
S12_230579178	P12	230,579,178	C/T	4.11E-08	7.39	2.45E-04	0.0593	0.2242	-0.4508
S12_230812962	P12	230,812,962	T/C	1.38E-08	7.86	1.21E-04	0.0579	0.2302	0.4852
S12_230887290	P12	230,887,290	C/G	3.02E-08	7.52	2.02E-04	0.0534	0.2259	-0.4727
S12_231022812	P12	231,022,812	G/T	5.21E-08	7.28	2.91E-04	0.0549	0.2229	-0.4674

*Position of detected SNPs in pepper reference genome version 1.6.

*Bold SNPs were commonly detected against KPC-7 and JHAI107 isolates.

Supplementary Table S5. Haplotype blocks estimated by genotyping-by-sequencing of the GWAS core collection.

Chromosome	Haplotype	Average size of LD blocks (kb)	Average number of SNPs per LD block
P1	3,267	76.4	15.6
P2	1,866	71.1	14.2
P3	2,723	83.0	15.7
P4	2,226	88.2	17.0
P5	2,321	84.5	17.2
P6	2,582	75.1	14.9
P7	2,548	81.2	16.9
P8	1,270	96.0	18.3
P9	2,623	84.3	16.7
P10	2,788	64.9	13.4
P11	2,953	72.6	16.8
P12	2,102	97.6	17.5
Total	29,269	81.2	16.2

Supplementary Table S6. Candidate genes located in the vicinity of the GWAS-SNPs and biparental QTLs and their functional annotations.

Attached as an excel file
